# Supplementary material for: Modeling the efficacy of CRISPR gene drive for snail immunity on schistosomiasis control
Source: PLoS Negl Trop Dis. 2022 Oct 31;16(10):e0010894. doi: 10.1371/journal.pntd.0010894 (PMC9648845; doi:10.1371/journal.pntd.0010894)
Supplement: S1 Text — Additional genetic results, including the evolution of resistance to a drive, daisy chain drives of 1–5 loci, and the sensitivity of GDMI to a variety of genetic variables. Additional epidemiological results, including invasion and extinction analyses as well as the sensitivity of GDMI to a variety of epidemiological variables. Fig A. Forward simulations under fixed epidemiological conditions of the spread of GDMI with various resistance production rates per homing event. (A) No resistant alleles are produced. (B) Resistant alleles are produced with 20% of homing events. GDMI achieves only low frequency in the population due to rapid evolution of resistance to the drive mechanism. (C) Resistant alleles are produced with 10% of homing events. GDMI rises slowly, achieving half the frequency in the population compared to conditions where resistance does not evolve. (D) Resistant alleles are produced with 10% of homing events as in panel C. In 20 years it is evident that the frequency of resistant alleles outpaces the homing efficiency benefits in inheritance of GDMI, and GDMI declines after reaching intermediate frequency (eventually to negligible frequency). Fig B. Forward simulations of daisy drive systems for the inheritance of GDMI designed with 1–5 daisy chain loci. Decay of the drive occurs after n generations, therefore more loci produce a longer lasting drive. However, because GDMI spreads slowly in the population compared to a fully outcrossed population, peak frequency of GDMI is low. Nearly 30 daisy chain loci are required to reach peak frequency of 50%, rendering daisy drive infeasible for implementation in this system. Fig C. The relationship between the immune and susceptible alleles described by the dominance coefficient governs the trajectory of evolution for naturally-occurring immunity. Lower dominance of the immune allele leads to slower evolution of immunity, which could change the speed at which GDMI increases in frequency in a population. Fig D. H [file pntd.0010894.s001.pdf]

# Supplementary Text: Modeling the efficacy of CRISPR gene drive for snail immunity on schistosomiasis control

Richard E. Grewelle[1,2*], Javier Perez-Saez[3], Josh Tycko[4], Erica K.O. Namigai[5], Chloe G. Rickards[6], Giulio A. De Leo[1,2,7*]

[1]Department of Biology, Stanford University, Stanford, CA, USA
[2]Hopkins Marine Station, Stanford University, Pacific Grove, CA, USA
[3]Department of Epidemiology, Johns Hopkins Bloomberg School of Public Health, Baltimore, MD, USA
[4]Department of Genetics, Stanford University, Stanford, CA, USA
[5]Department of Zoology, University of Oxford, Oxford, UK
[6]Department of Biology, University of California Santa Cruz, Santa Cruz, CA, USA
[7]Woods Institute for the Environment, Stanford University, Stanford, CA, USA
*To whom correspondence should be addressed;
E-mail: regrew@stanford.edu, deleo@stanford.edu

## Population genetic model

The evolution of a focal population seeded with gene drive mediated immune (GDMI) snails is described in rudimentary form in the model summary with transition matrix $\boldsymbol{Q}$. This describes a system of inheritance where a susceptible and immune allele are present in the population, and the GDMI allele exhibits the same immunity as the naturally occurring immunity. The three alleles form six distinct genotypes in a diploid species. $\boldsymbol{Q}$ represents random mating with no selection and full population replacement each generation. In a natural system assortative mating, selection, and iteroparity are known to occur. Because assortative mating as a function of innate immunity to schistosome infection has not yet been demonstrated in host snail species,

we maintain this assumption in our population genetic model. However, because several modes of selection are described for this immunity and iteroparity produces overlapping generations, other assumptions for $\boldsymbol{Q}$ must be relaxed to accurately reflect the evolutionary dynamics of the snails. Viability and fecundity selection are separately accounted in their contribution the fitness of each genotype, as their relative importance in determining the rate of evolution changes with the model of population dynamics and replacement.

## Birth-death process

Snail recruitment is density-dependent. Adult lifespan extends past the mean generation time, allowing for nearly continuous reproduction after sexual maturity. We assume that background mortality is density-independent and that the population replacement rate is modulated by changes in mortality rate provided density-dependent recruitment is sufficient to for full replacement. In contrast to a population model described by reproduction proceeded by culling to a carrying capacity, we model snail population dynamics with culling proceeded by reproduction to a carrying capacity. The sub-population size of genotype $i$ is described through time with the two step process: (1) death and migration yield the reproducing population of genotype $i$, $\bar{N}_i(t)$, which (2) give birth to offspring according to equation 9.

$$\bar{N}_i(t) = N_i(t)[1 - \gamma_i(t) + m_i(t)] \tag{8}$$

$$N_i(t+1) = \bar{N}_i(t) + \frac{\lambda_i(t)}{\lambda(t)}[G(\bar{N}(t), \lambda(t), t) - \bar{N}(t)] \tag{9}$$

where $N_i(t)$ is the genotype sub-population size in generation $t$, $\gamma_i(t)$ is the fractional mortality in generation $t$, $m_i(t)$ is the fractional net migration in or out of the focal population in generation $t$, $\lambda_i(t)$ is the partial finite growth of genotype $i$ after mortality and migration, and $\lambda(t)$ is the maximum total finite growth of the population. Because deaths are separately

accounted in this birth-death process, here $\lambda(t)$ resembles fecundity (i.e. when mortality is absent, fecundity and finite population growth are equivalent). $G(\bar{N}(t), \lambda(t), t)$ is the discrete time population growth function which describes total population growth. We use a logistic growth function in the simulations throughout the paper.

$$G(\bar{N}(t), \lambda(t), t) = \frac{\bar{N}(t)K}{\bar{N}(t) + (K - \bar{N}(t))e^{-\lambda(t)}} \tag{10}$$

## Viability selection

Viability selection on immunity to schistosome infection is incorporated in the fractional mortality term, $\gamma_i(t)$, which is a function of background mortality (neutral) and loss from the reproductive population through infection (directional selection), which has been demonstrated to substantially increase mortality and castrate snails. For these two reasons, we assume that infected snails are removed from the reproducing population. Reproductive compensation has been observed for snails exposed to miracidia, whereby these snails produce offspring at higher rates following exposure. This could counter lost reproduction after infection. However, a genetic link between reproductive compensation and immunity has not yet been shown. Therefore, we assume that the mechanism of reproductive compensation is phenomenological, being linked to exposure but not patent infection, rather than linked to host genetics, and does not produce fitness differences between genotypes. Explicitly, we write $\gamma_i(t)$ as

$$\gamma_i(t) = d + (1 - d)Pr(y^+)(1 - h\iota) \tag{11}$$

$d$ is the adult background mortality per snail in a generation. $Pr(y^+)$ is the per snail probability of acquiring a new infection. $h$ is the dominance coefficient, which takes values between 0 and 1, with a value of 0.5 indicating co-dominance and a value of 1 indicating complete dominance of the immune allele over the susceptible allele. $\iota \in [0, 1]$ is the degree of immunity

conferred by the immune allele compared to the susceptible allele. Genetically, this value can be equated to the penetrance of innate immunity. A value of 0 indicates no additional immunity, while a value of 1 indicates full immunity. The loss of reproductive individuals from the population contributed by infection compared to the background mortality determines the strength of viability selection for immunity in the population.

## Fecundity selection

Inbreeding and immunity are known to negatively impact reproductive success in host snail populations. Inbreeding can reduce fecundity and egg viability, while immunity is associated with low egg viability [1, 2]. Because the population model tracks reproductive individuals, and recruitment of offspring to the reproductive class is density-dependent, offspring viability can be treated as a component of fecundity. Using the broad definition of fecundity as the offspring surviving to adulthood, we institute fecundity costs for inbreeding by self-fertilization and for maintenance of immune alleles. Cost of immune maintenance, $C$, is directly related to the phenotype and is dose-independent (i.e. 2 immune alleles are not more costly than 1 immune allele with full dominance). An additional cost, $C_g$, is associated with maintenance of the genetic payload in the drive construct. This cost is dose-dependent; gene drive homozygotes carry a two-fold cost compared to heterozygotes. Let the set of alleles $\{A, B, B_g\}$ be indexed as $\{1, 2, 3\}$. The fecundity of each genotype, $f_i$, can be represented as:

$$f_{11} = f_{AA} \tag{12}$$

$$f_{12} = f_{11}(1 - hC) \tag{13}$$

$$f_{22} = f_{11}(1 - C) \tag{14}$$

$$f_{13} = f_{11}(1 - hC - C_g) \tag{15}$$

$$f_{23} = f_{11}(1 - C - C_g) \tag{16}$$

$$f_{33} = f_{11}(1 - C - 2C_g) \tag{17}$$

The inbreeding cost is not directly associated with a specific genotype, but rather is incorporated as $\xi \in [0, 1]$ in the calculation of $\boldsymbol{Q}$ (equation 3):

$$\boldsymbol{Q} = \sigma(1 - \xi)\boldsymbol{S} + (1 - \sigma)\boldsymbol{T} \tag{18}$$

Although $\xi$ is a cost not directly applied to any genotype, because it is a cost of inbreeding due to self-fertilization and self-fertilization produces homozygotes with higher frequency than outcrossing, this cost reduces the fitness of homozygotes relative to heterozygotes when selfing is common in the population. Inbreeding is assumed to only affect the F1 generation of selfing parents, and associated costs are not separately tracked through descent in future generations. This treatment is reasonable for a sufficient degree of outcrossing which mixes lineages in the population. Highly inbred snail populations are shown to be insensitive to inbreeding depression caused by selfing, presumably due to purging of deleterious alleles from the gene pool, and therefore inbreeding costs are low in the absence of outcrossing. Table A gives parameter values used in the genetic model, many of which are known to vary by species or even by population and environmental conditions. Values were chosen to be centered in the range of observed values with references given to empirical measurements. Results can differ by system, and the

Table A: Default parameter values for the genetic model

| Parameter | Description | Value | Ref. |
|---|---|---|---|
| $d$ | background mortality per generation | 0.5 | calculated with reference to *B. pfeifferi* [3, 4] |
| $h$ | dominance coefficient for immune allele | 1 | supported by Fig 2 in main text and [5] |
| $Pr(y^{+})$ | probability of infection per generation | 0.15 | derived from epidemiological model (equn. 90) |
| $\iota$ | penetrance of immunity | 0.8 | [1] |
| $C$ | cost of immunity | 0.2835 | model-derived to yield symmetric selection for and against immunity, stabilizing the phenotypic ratio at 1:1 |
| $C_g$ | cost of payload per copy | 0.1 | [6] variable/theoretical |
| $\xi$ | cost of inbreeding | 0.3 | [7] highly variable |
| $H$ | homing efficiency | 0.9 | [8] |
| $\sigma$ | selfing frequency | 0.5 | [7] highly variable |
| $f_{AA}$ | per generation fecundity of a susceptible snail | 20 | [1] |
| $P_{AA}(t=0)$ | natural initial frequency of susceptible genotype | 0.5 | [1, 5] |
| $P_{A}(t=0)$ | natural initial frequency of allele A | $\frac{\sigma-\sqrt{16P_{AA}-24\sigma P_{AA}+\sigma^2(1+8P_{AA}))}}{4(\sigma-1)}$ | calculated for a given $\sigma$ |
| $P_{B}(t=0)$ | natural initial frequency of allele B | 1-$P_A$ | calculated for a given $\sigma$ |
| $P_{AB}(t=0)$ | natural initial frequency of heterozygote | $\frac{4P_AP_B(1-\sigma)}{2-\sigma}$ | calculated for a given $\sigma$ |
| $P_{BB}(t=0)$ | natural initial frequency of immune genotype | $\frac{P_B^2+P_AP_B\sigma}{2-\sigma}$ | calculated for a given $\sigma$ |

endpoint sensitivity analyses in Figs 3 and 4 (main text) provide indication of the most sensitive parameters.

The Markov process described in the model summary with equations 3 and 4 represents a semelparous population that reproduces once per generation, and adults are completely replaced by offspring with no consideration for fitness differences between genotypes. Instead, however, snail host populations reproduce continuously with overlapping generations. We devise a modified Markov process to describe these evolutionary dynamics, incorporating the fitness differences detailed above in equations 12-17. We consider the modified transition matrices:

$$
\bar{\mathbf{S}} = \begin{pmatrix}
f_{11} & 0 & 0 & 0 & 0 & 0 \\
\frac{f_{12}}{4} & \frac{f_{12}}{2} & \frac{f_{12}}{4} & 0 & 0 & 0 \\
0 & 0 & f_{22} & 0 & 0 & 0 \\
\frac{f_{13}}{4} & 0 & 0 & \frac{f_{13}(1-H)}{2} & 0 & f_{13}(\frac{H}{2} + \frac{1}{4}) \\
0 & 0 & \frac{f_{23}}{4} & 0 & \frac{f_{23}(1-H)}{2} & \frac{f_{23}H}{2} + \frac{f_{23}}{4} \\
0 & 0 & 0 & 0 & 0 & f_{33}
\end{pmatrix} \tag{19}
$$

$$
\bar{\mathbf{T}} = \begin{pmatrix}
a_{11} & a_{12} & 0 & a_{14} & 0 & a_{16} \\
a_{21} & a_{22} & a_{23} & a_{24} & a_{25} & a_{26} \\
0 & a_{32} & a_{33} & 0 & a_{35} & a_{36} \\
a_{41} & a_{42} & 0 & a_{44} & a_{45} & a_{46} \\
0 & a_{52} & a_{53} & a_{54} & a_{55} & a_{56} \\
0 & 0 & 0 & a_{64} & a_{65} & a_{66}
\end{pmatrix} \tag{20}
$$

$$a_{11} = f_{11}P_{11} + \frac{(f_{11} + f_{12})P_{12} + (f_{11} + f_{13})P_{13}}{4} \tag{21}$$

$$a_{12} = \frac{(f_{11} + f_{22})P_{22}}{2} + \frac{(f_{11} + f_{12})P_{12} + (f_{11} + f_{23})P_{23}}{4} \tag{22}$$

$$a_{14} = (1 - H)(\frac{(f_{11} + f_{33})P_{33}}{2} + \frac{(f_{11} + f_{13})P_{13} + (f_{11} + f_{23})P_{23}}{4}) \tag{23}$$

$$a_{16} = H(\frac{(f_{11} + f_{33})P_{33}}{2} + \frac{(f_{11} + f_{13})P_{13} + (f_{11} + f_{23})P_{23}}{4}) \tag{24}$$

$$a_{21} = \frac{(f_{11} + f_{12})P_{11}}{4} + \frac{2f_{12}P_{12} + (f_{12} + f_{13})P_{13}}{8} \tag{25}$$

$$a_{22} = \frac{(f_{11} + f_{12})P_{11} + 2f_{12}P_{12} + (f_{12} + f_{22})P_{22}}{4} + \frac{(f_{12} + f_{13})P_{13} + (f_{12} + f_{23})P_{23}}{8} \tag{26}$$

$$a_{23} = \frac{(f_{12} + f_{22})P_{22}}{4} + \frac{2f_{12}P_{12} + (f_{12} + f_{23})P_{23}}{8} \tag{27}$$

$$a_{24} = (1 - H)(\frac{(f_{12} + f_{33})P_{33}}{4} + \frac{(f_{12} + f_{13})P_{13} + (f_{12} + f_{23})P_{23}}{8}) \tag{28}$$

$$a_{25} = (1 - H)(\frac{(f_{12} + f_{33})P_{33}}{4} + \frac{(f_{12} + f_{13})P_{13} + (f_{12} + f_{23})P_{23}}{8}) \tag{29}$$

$$a_{26} = H(\frac{(f_{12} + f_{33})P_{33}}{2} + \frac{(f_{12} + f_{13})P_{13} + (f_{12} + f_{23})P_{23}}{4}) \tag{30}$$

$$a_{32} = \frac{(f_{11} + f_{22})P_{11}}{2} + \frac{(f_{22} + f_{12})P_{12} + (f_{22} + f_{13})P_{13}}{4} \tag{31}$$

$$a_{33} = f_{22}P_{22} + \frac{(f_{22} + f_{12})P_{12} + (f_{22} + f_{23})P_{23}}{4} \tag{32}$$

$$a_{35} = (1 - H)(\frac{(f_{22} + f_{33})P_{33}}{2} + \frac{(f_{22} + f_{13})P_{13} + (f_{22} + f_{23})P_{23}}{4}) \tag{33}$$

$$a_{36} = H(\frac{(f_{22} + f_{33})P_{33}}{2} + \frac{(f_{22} + f_{13})P_{13} + (f_{22} + f_{23})P_{23}}{4}) \tag{34}$$

$$a_{41} = \frac{(f_{11} + f_{13})P_{11}}{4} + \frac{(f_{12} + f_{13})P_{12} + 2f_{13}P_{13}}{8} \tag{35}$$

$$a_{42} = \frac{(f_{13} + f_{22})P_{22}}{4} + \frac{(f_{12} + f_{13})P_{12} + (f_{13} + f_{23})P_{23}}{8} \tag{36}$$

$$a_{44} = (1 - H)(\frac{(f_{13} + f_{11})P_{11} + 2f_{13}P_{13} + (f_{13} + f_{33})P_{33}}{4} + \frac{(f_{13} + P_{12})P_{12} + (f_{13} + f_{23})P_{23}}{8}) \tag{37}$$

$$a_{45} = (1 - H)(\frac{(f_{13} + f_{22})P_{22}}{4} + \frac{(f_{12} + f_{13})P_{12} + (f_{13} + f_{23})P_{23}}{8}) \tag{38}$$

$$a_{46} = H(\frac{(f_{13}+f_{11})P_{11} + (f_{13}+f_{12})P_{12} + (f_{13}+f_{22})P_{22} + 2f_{13}P_{13} + (f_{13}+f_{23})P_{23} + (f_{13}+f_{33})P_{33}}{4})+ \tag{39}$$

$$\frac{2(f_{13}+f_{33})P_{33} + 2f_{13}P_{13} + (f_{23}+f_{13})P_{23}}{8} \tag{40}$$

$$a_{52} = \frac{(f_{11}+f_{23})P_{11}}{4} + \frac{(f_{12}+f_{23})P_{12} + (f_{23}+f_{13})P_{13}}{8} \tag{41}$$

$$a_{53} = \frac{(f_{23}+f_{22})P_{22}}{4} + \frac{(f_{23}+f_{12})P_{12} + 2f_{23}P_{23}}{8} \tag{42}$$

$$a_{54} = (1-H)(\frac{(f_{11}+f_{23})P_{11}}{4} + \frac{(f_{12}+f_{23})P_{12} + (f_{23}+f_{13})P_{13}}{8}) \tag{43}$$

$$a_{55} = (1-H)(\frac{(f_{23}+f_{22})P_{22} + (f_{23}+f_{33})P_{33} + 2f_{23}P_{23}}{4} + \frac{(f_{23}+f_{12})P_{12} + (f_{23}+f_{13})P_{13}}{8}) \tag{44}$$

$$a_{56} = H(\frac{(f_{23}+f_{11})P_{11} + (f_{23}+f_{12})P_{12} + (f_{23}+f_{22})P_{22} + (f_{13}+f_{23})P_{13} + 2f_{23}P_{23} + (f_{23}+f_{33})P_{33}}{4})+ \tag{45}$$

$$\frac{2(f_{23}+f_{33})P_{33} + (f_{13}+f_{23})P_{13} + 2f_{23}P_{23}}{8}$$

$$a_{64} = (1-H)(\frac{(f_{11}+f_{33})P_{11}}{2} + \frac{(f_{33}+f_{12})P_{12} + (f_{33}+f_{13})P_{13}}{4}) \tag{46}$$

$$a_{65} = (1-H)(\frac{(f_{22}+f_{33})P_{22}}{2} + \frac{(f_{33}+f_{12})P_{12} + (f_{33}+f_{23})P_{23}}{4}) \tag{47}$$

$$a_{66} = \frac{H}{2}((f_{11}+f_{33})P_{11} + (f_{12}+f_{33})P_{12} + (f_{22}+f_{33})P_{22} + \frac{(f_{13}+f_{33})P_{13}}{2} + \frac{(f_{23}+f_{33})P_{23}}{2})+ \tag{48}$$

$$\frac{(f_{13}+f_{33})P_{13} + (f_{23}+f_{33})P_{23}}{4} + f_{33}P_{33}$$

Modifying the equation for $\boldsymbol{Q}$ in the model summary to reflect the incorporation of demography and fitness differences between genotypes, we achieve:

$$\bar{\boldsymbol{Q}} = \sigma(1-\xi)\bar{\boldsymbol{S}} + (1-\sigma)\bar{\boldsymbol{T}} \tag{49}$$

The vector of genotype frequencies at time $t$ is denoted $\boldsymbol{P}(t)$. $P_i(t)$ represents the frequency of genotype i at time $t$. The vector of partial growth rates is $\boldsymbol{\lambda}(t)$:

$$\boldsymbol{\lambda}(t) = \boldsymbol{P}(t)\bar{\boldsymbol{Q}}(t) \tag{50}$$

and the sum of the elements of this vector is $\lambda(t)$:

$$\lambda(t) = \sum_{i=1}^{6} \lambda_i(t) \tag{51}$$

These values can be substituted in equation 9 to track the evolution of the population.

# Establishing initial genetic conditions

Prior to deploying GDMI snails in a naive population, the standing background genetic variation for susceptibility to infection has some influence over the success of GDMI. The two forms of genetic variation that are important to consider are: the frequency of susceptibility and the distribution of the susceptible allele across the genotypes. High self-fertilization frequencies favor homozygous populations, which exposes the susceptible allele to selection (assuming it is recessive). Several studies have measured susceptibility empirically through challenge experiments in laboratory conditions. Snail populations that are not far removed from a natural parental lineage demonstrate intermediate levels of susceptibility, though these results vary with miracidial dosing. For simplicity we fix the standing natural frequency of susceptibility at 0.5. The immune phenotype is, therefore, at 0.5 frequency as well in our idealized starting conditions. In a mixed-mating system, which these snails exhibit, the distribution of the $A$ and $B$ alleles across the three genotypes is modulated by selfing frequency. The transition matrix describing the evolution of the three naturally occurring genotypes is

$$\mathbf{Q}_{natural} = \begin{pmatrix} \sigma + (1-\sigma)P_A & (1-\sigma)P_B & 0 \\ \frac{\sigma}{4} + \frac{(1-\sigma)P_A}{2} & \frac{\sigma}{2} + \frac{(1-\sigma)(P_A+P_B)}{2} & \frac{\sigma}{4} + \frac{(1-\sigma)P_B}{2} \\ 0 & (1-\sigma)P_A & \sigma + (1-\sigma)P_B \end{pmatrix} \tag{52}$$

Here we denote $P_A$ and $P_B$ as the allele frequencies, and $P_{11}(t)$, $P_{12}(t)$, $P_{22}(t)$ as the genotype frequencies at time $t$. To establish initial genetic conditions, we find the equilibrium genotype frequencies given a frequency of self-fertilization. We assume that the genotype frequencies are the result of selection but that otherwise selection is not stronger than the equilibrium behavior of the transition matrix under neutral conditions. Therefore, genotype frequencies can be solved given the frequency of the susceptible genotype. Equilibrium behavior of this transition matrix is strong, with genotype frequencies approaching equilibrium geometrically so that equilibrium is effectively reached within 2 generations. In the absence of imposed selection, the allele frequencies remain constant through each generation despite the changing genotype frequencies. This is the reason genotype frequencies are represented as functions of time above, while allele frequencies are not. From matrix multiplication, we know the frequency of the susceptible genotype in the next generation using $\mathbf{Q}_{natural}$ is:

$$\begin{aligned} P_{11}(t+1) &= P_{11}(t)(\sigma + (1-\sigma)P_A) + P_{12}(t)(\frac{\sigma}{4} + \frac{(1-\sigma)P_A}{2}) \\ &= (1-\sigma)P_A^2 + \sigma(P_{11}(t) + \frac{P_{12}(t)}{4}) \\ &= P_A^2 + \sigma P_A P_B - \frac{\sigma P_{12}(t)}{4} \end{aligned} \tag{53}$$

Solving the difference equation yields:

$$P_{11}(t+1) = P_A^2 + \sigma P_A P_B(1 - \frac{1-\sigma}{2-\sigma}(1 - (\frac{\sigma}{2})^t)) - \frac{\sigma P_{12}(0)}{4}(\frac{\sigma}{2})^t \tag{54}$$

The limiting distribution of genotype frequencies can be solved as $t \to \infty$. For the susceptible genotype, this gives:

$$P_{11}(\infty) = P_A^2 + \frac{\sigma P_A P_B}{2-\sigma} \tag{55}$$

The same derivations can be performed for the other two genotype frequencies from the transition matrix to achieve the limiting distribution.

$$\begin{aligned} P_{12}(t+1) &= P_{11}(t)(1-\sigma)P_B + P_{12}(t)(\frac{\sigma}{2} + \frac{(1-\sigma)(P_A+P_B)}{2}) + P_{22}(t)(1-\sigma)P_A \quad (56) \\ &= 2(1-\sigma)P_AP_B + \frac{\sigma P_{12}(t)}{2} \\ P_{22}(t+1) &= P_{12}(t)(\frac{\sigma}{4} + \frac{(1-\sigma)P_B}{2}) + P_{22}(t)(\sigma + (1-\sigma)P_B) \quad (57) \\ &= (1-\sigma)P_B^2 + \sigma(P_{22}(t) + \frac{P_{12}(t)}{4}) \end{aligned}$$

The equilibrium values for the heterozygote and immune homozygote are:

$$P_{12}(\infty) = \frac{4P_AP_B(1-\sigma)}{2-\sigma} \quad (58)$$

$$P_{22}(\infty) = \frac{P_B^2 + P_AP_B\sigma}{2-\sigma} \quad (59)$$

These results differ from the results presented by Karlin [9] due to a presumed typographical error in his text. A population with 50% susceptible genotype at equilibrium is assumed when GDMI snails are introduced (simulation $t = 0$). Given that $P_{11}(t = 0) = 0.5$, $P_A$ can be solved:

$$P_B = 1 - P_A \quad (60)$$

$$P_{11}(t=0) = P_A^2 + \frac{\sigma P_A(1-P_A)}{2-\sigma} \quad (61)$$

$$\begin{aligned} P_A &= \frac{\sigma - \sqrt{16P_{11}(t=0) - 24\sigma P_{11}(t=0) + \sigma^2(1+8P_{11}(t=0)))}}{4(\sigma-1)} \quad (62) \\ P_A|_{P_{11}=0.5} &= \frac{\sigma - \sqrt{8 - 12\sigma + 5\sigma^2}}{4(\sigma-1)} \end{aligned}$$

## Evolution of resistance

Resistance to the drive mechanism can readily develop if the target sequence on the homologous chromosome is mutated so as to be unrecognizable by guide RNA. This occurs primarily through non-homologous end joining (NHEJ), which is an alternative mechanism of double strand break repair that can occur instead of homology directed repair. Point mutations and standing genetic variation at the target locus can also result in resistance to the drive mechanism. Because NHEJ is a common repair pathway in most organisms, it is the primary producer of resistance in the population, especially as the drive construct increases in frequency in the population. Resistance formation via this pathway occurs due to misrepair after cleavage from the Cas nuclease and occurs proportionally to the number of cleavage events that occur in the population each generation. Homing efficiency is a function of the predominance of homology directed repair over NHEJ, though not every failed drive event is due to NHEJ. Homing efficiency at the population level declines as resistant alleles accumulate. Resistant alleles may represent a spectrum of mutations, and separately accounting for the variety of alleles and their respective fitness requires exponential expansion of the number of genotypes tracked in this genetic model. To simplify, we assume that resistant alleles are equivalent in fitness to their natural counterparts. This gives them a fecundity advantage to the drive allele, which carries an additional cost due to the genetic payload. In a randomly mating population, outcrossing events will randomly pair resistant alleles with each other, natural alleles, or the drive allele. The consequence of non-assortative mating is that the formation of resistant alleles is proportional to the number of gene drive heterozygotes produced due to failed homing. The number of gene drive heterozygotes (hybrids) produced each generation is:

$$N_{hybrids}(t) = \frac{\lambda_4(t) + \lambda_5(t)}{\lambda(t)}[G(\bar{N}(t), \lambda(t), t) - \bar{N}(t)] \tag{63}$$

The homing efficiency in generation $t$ can be calculated from the maximum homing efficiency without NHEJ, $H_0$, at $t = 0$ when resistant allele accumulation is lowest and determined only by background resistance due to standing genetic variation.

$$\begin{aligned} H(t) &= H_0(1 - R(t) - \nu(1 - R(t))) \\ &= H_0(1 - R(t))(1 - \nu) \end{aligned} \tag{64}$$

$R(t)$ is the frequency of resistant alleles in the pool of natural and resistant alleles (excluding the drive allele). $\nu$ is the per homing event rate of production of resistant alleles. If the population is in mutation-selection-drift balance, the rate of production is almost entirely due to NHEJ. The rate of accumulation of resistant alleles is the fraction of the total gene drive heterozygotes (hybrids) produced that are resistant multiplied by the fraction of hybrids produced in the total population each generation.

$$\begin{aligned} R(t+1) &= R(t) + \frac{\nu(1 - R(t))N_{hybrids}(t)}{(1 - H(t) + \nu(1 - R(t)))N(t)} \\ &= R(t) + \frac{\nu(1 - R(t))N_{hybrids}(t)}{(1 - H_0(1 - R(t))(1 - \nu) + \nu(1 - R(t)))N(t)} \\ &= R(t) + \frac{\nu(1 - R(t))N_{hybrids}(t)}{(1 - (1 - R(t))(H_0(1 - \nu) + \nu)N(t)} \end{aligned} \tag{65}$$

Should mutations leading to resistance be deleterious compared to the respective naturally occurring alleles, resistance is expected to spread more slowly in the population than is depicted in Fig A. Conversely, should mutations be beneficial, spread will occur more rapidly. The cost of resistance can be modulated by choosing neutral regions for lower cost and regions under strong selection for higher cost (e.g. ribosomal RNA genes). Fitness costs due to off-target CRISPR-induced mutations are possible, but experimental evidence indicates that the frequency of these mutations is low (<2%). Moreover, these mutations do not exhibit drive

and are not related to the evolution of resistance. Off-target mutations can be reduced with properly specific gRNA design and restricted expression of the CAS9 protein. The influence of resistance evolution on the establishment of GDMI is explored in Fig A.

## Daisy chain loci

Previous authors have proposed mechanisms to safeguard the spread and persistence of gene drives in wild populations. One prominent mechanism is known as a daisy drive, which borrows its name from the concept of a daisy chain in which elements are connected in a series. Daisy drives are split drives which split the drive element from the payload by positioning them on separate loci, ideally on different chromosomes for independent inheritance. Daisy drives incorporate multiple splits, with one drive element necessary to produce super-Mendelian inheritance of the next element in the chain. The base of the chain is a non-drive element and the tip of the chain is the genetic payload containing the gene of interest to be inherited in the population. Each element in the chain increases in frequency temporarily and then decays as the preceding elements decline in frequency in the population through natural selection. Each element in the daisy chain is considered haplosufficient, so one copy produces the intended homing efficiency of the proceeding element. Because daisy chain elements (loci) are introduced together in engineered individuals, spread of the payload gene occurs locally within a lineage in association with other loci and cannot be modeled using the same framework so far introduced which describes random pairing of alleles from one locus. The homing efficiency associated with the payload locus is primarily determined by this local lineage-based process of inheritance of daisy chain loci, especially when loci are at low frequency, and secondarily through outcrossing events with other lineages in the population that maintain daisy chain loci. The secondary process becomes consequential at high frequencies of daisy chain loci in the population. Homing efficiency of the payload corresponds is determined by the frequency of

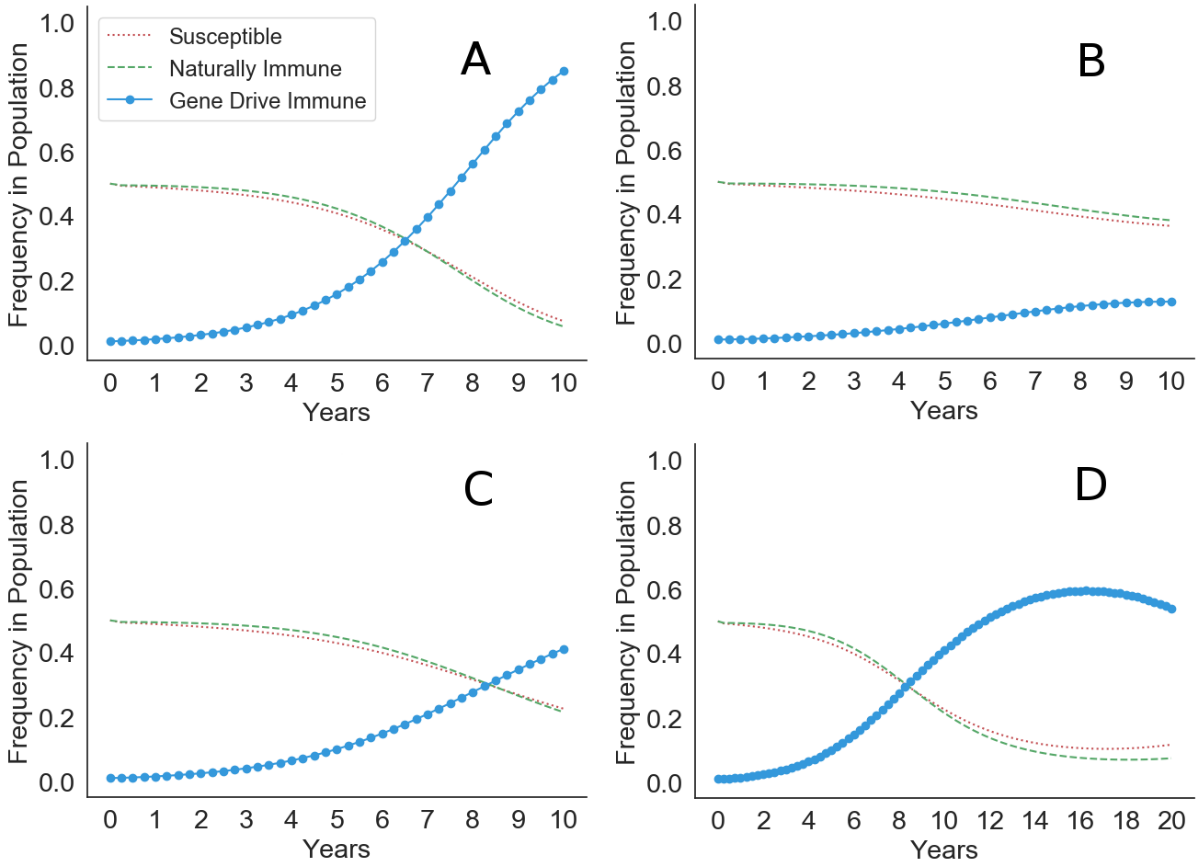


Figure A: Forward simulations under fixed epidemiological conditions of the spread of GDMI with various resistance production rates per homing event. (A) No resistant alleles are produced. (B) Resistant alleles are produced with 20% of homing events. GDMI achieves only low frequency in the population due to rapid evolution of resistance to the drive mechanism. (C) Resistant alleles are produced with 10% of homing events. GDMI rises slowly, achieving half the frequency in the population compared to conditions where resistance does not evolve. (D) Resistant alleles are produced with 10% of homing events as in panel C. In 20 years it is evident that the frequency of resistant alleles outpaces the homing efficiency benefits in inheritance of GDMI, and GDMI declines after reaching intermediate frequency (eventually to negligible frequency).

the preceding locus in the daisy chain. We set $\delta(t)$ as the daisy chain coefficient modulating the homing efficiency. The daisy coefficient is directly proportional to the co-occurrence of the payload and the preceding drive element and can be calculated as follows through time for lineage-specific inheritance (i.e. each outcrossing event occurs with a snail with no drive alleles):

$$lim\ inf\ \delta(t) = \begin{cases} 1 & n = 0 \\ 2^{1-t} & n = 1 \\ \frac{2^{n-1}+2^{n-2}(t-n)}{2^{t-1}} & n > 1 \end{cases} \tag{66}$$

$n$ is the number of splits in the drive design, which is equivalent to the number of drive elements in the daisy chain (excluding the payload). This is the lower limit for the value of $\delta(t)$, as homing efficiency for the payload increases with the accumulation of the preceding drive element in the population so that with each outcrossing event, the mate outside the primary lineage may carry the preceding drive element. The frequency of the preceding drive element in the population is always lower than the frequency of the payload, and is the frequency of the payload in the prior generation before the peak frequency of the payload is reached. Fitness costs of each drive element are assumed negligible compared to the cost of the genetic payload at the tip of the daisy chain. After the peak frequency is reached, the frequency of the preceding drive element is lower than the frequency of the payload in the prior generation. We can state the following:

$$lim\ sup\ \delta(t) = \begin{cases} 1 & n = 0 \\ 2^{1-t} & n = 1 \\ \frac{2^{n-1}+2^{n-2}(t-n)}{2^{t-1}} + P_{payload}(t-1) & n > 1 \end{cases} \tag{67}$$

$$\delta(t) = \begin{cases} 1 & n = 0 \\ 2^{1-t} & n = 1 \\ \frac{2^{n-1}+2^{n-2}(t-n)}{2^{t-1}} + P_{drive}(t) & n > 1 \end{cases} \tag{68}$$

where $P_{payload}(t)$ and $P_{drive}(t)$ are the frequencies of the genetic payload and the associated

preceding drive element in the daisy chain, respectively. $lim\ sup\ \delta(t) = \delta(t)$ prior to peak payload frequency. Equation 67 is useful to calculate the peak frequency of the payload in a daisy drive system without the need for a system of equations for each locus, which quickly becomes complex with additional elements in the chain. If we consider $H_0$ as the maximum homing efficiency without a daisy chain design (1 locus, $n = 0$), then the observed homing efficiency through time can be calculated as:

$$H(t) = \delta(t)H_0 \tag{69}$$

The homing efficiency at time $t$ above represents the calculation for non-overlapping generations where the population is fully replaced with offspring each generation. When generations overlap, this homing efficiency underestimates the observed homing efficiency because younger adults reproduce alongside older adults, which maintain higher loads of daisy chain loci from fewer outcrossing events. Older adults produce more GDMI offspring as a result. The observed homing efficiency at the population level at time $t$ is a function of the survival of each age class. We calculate the surviving fraction of the payload allele as:

$$S_{B_g}(t) = \frac{\bar{N}_4(t) + \bar{N}_5(t) + 2\bar{N}_6(t)}{2} \tag{70}$$

Let $\boldsymbol{S_{B_g}}$ be the vector of surviving fractions of the payload allele through time.

$$\boldsymbol{S_{B_g}} = [S_{B_g}(0), S_{B_g}(1), \ldots, S_{B_g}(t)] \tag{71}$$

An age distribution, $\boldsymbol{\bar{Z}_{B_g}}$, can be produced by calculating the survival of each age class through time and normalizing the vector by the sum of the elements.

$$\boldsymbol{Z_{B_g}} = [S_{B_g}(0), \prod_{i=0}^{1} S_{B_g}(i), \frac{\prod_{i=0}^{2} S_{B_g}(i)}{\prod_{i=0}^{1} S_{B_g}(i)}, \ldots, \frac{\prod_{i=0}^{t} S_{B_g}(i)}{\prod_{i=0}^{t-1} S_{B_g}(i)}] \tag{72}$$

$$\boldsymbol{\bar{Z}_{B_g}} = \frac{\boldsymbol{Z_{B_g}}}{\sum_i Z_{B_g}(i)} \tag{73}$$

An adjusted daisy chain coefficient, $\delta_{adj}(t)$, can be produced by calculating the dot product of the vector of daisy chain coefficients and the age distribution:

$$\delta_{adj}(t) = \boldsymbol{\delta} \boldsymbol{\bar{Z}_{B_g}}^T \tag{74}$$

Homing efficiency at time $t$ for a population with overlapping generations is therefore:

$$H(t) = \delta_{adj}(t) H_0 \tag{75}$$

This value for the homing efficiency in each generation can be substituted into the existing framework described to calculate the frequency of GDMI through time. Fig B shows the trajectory of GDMI with the use of an increasing number of daisy chain loci.

## Dominance and penetrance of immune allele

The simulation in Fig 2 (main text) demonstrates a strong qualitative fit to empirical data from selection experiments. The frequency of infection, which is the fraction of infected snails out of the total surviving exposed individuals, is a phenotype resulting from immunity to a miracidial strain. The phenotype is a function of the exposure dose (number of miracidia) and the genetic underpinnings of immunity, including the number of loci involved, the dominance of immune alleles over susceptible alleles, and the penetrance of immune alleles. The genetic contributions to this phenotype could be myriad, but some large-effect loci have been identified in model snail species like *Biomphalaria glabrata*. These loci tend to be regions with high genetic variability and are linked to transmembrane proteins and receptors, which suggests a role in epitope

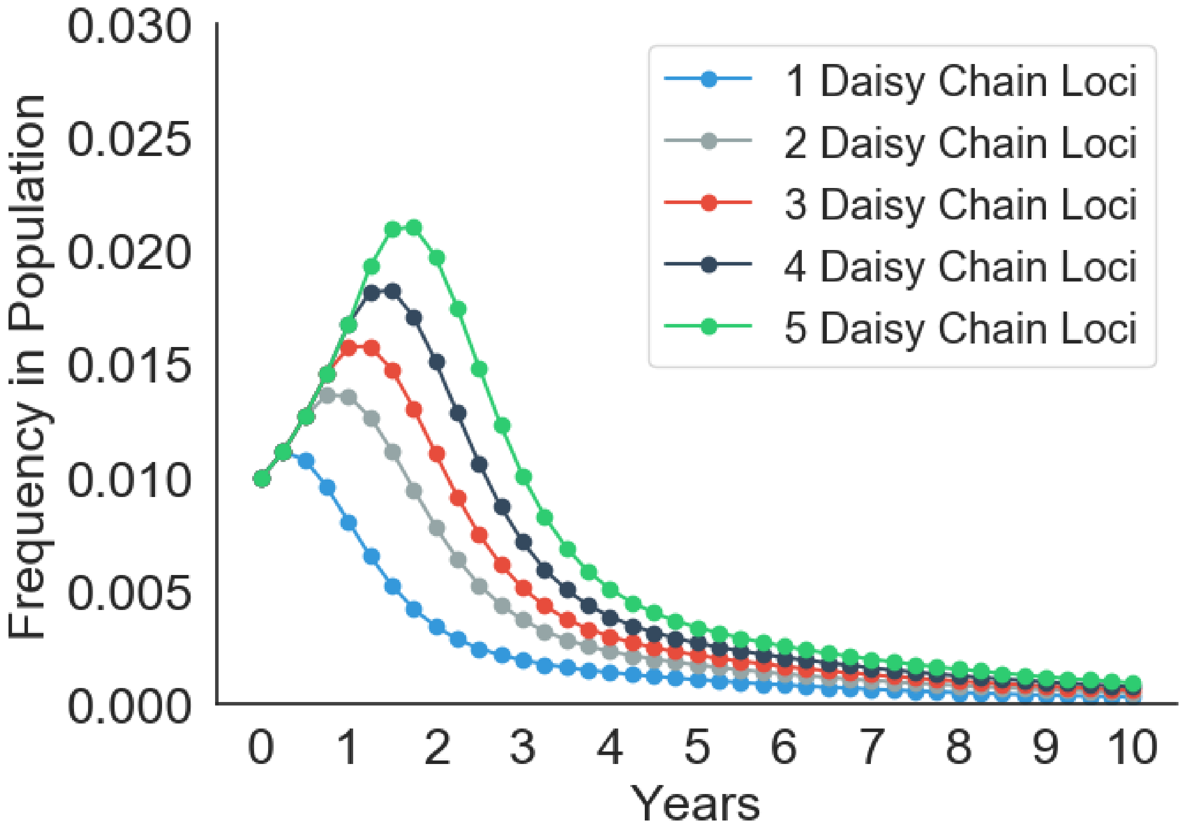


Figure B: Forward simulations of daisy drive systems for the inheritance of GDMI designed with 1-5 daisy chain loci. Decay of the drive occurs after $n$ generations, therefore more loci produce a longer lasting drive. However, because GDMI spreads slowly in the population compared to a fully outcrossed population, peak frequency of GDMI is low. Nearly 30 daisy chain loci are required to reach peak frequency of 50%, rendering daisy drive infeasible for implementation in this system.

recognition of an invading miracidium or sporocyst. One large-effect locus identified in Tennessen et al. 2015 served as a template for the default parameters used in the simulations in this work. We assumed a single locus model to represent this tightly linked gene cluster, and dominance of the immune allele over the susceptible allele was assumed complete as demonstrated in their empirical work. With a penetrance of 0.8, the model closely replicated observed evolution of immunity. However, as genetic work, such as genome wide association studies, identify new regions associated with immunity to schistosome infection in snails, more clarity will exist in the genetic contributions to immunity. Genes conferring immunity to one species or strain of schistosome may not confer immunity to others. These genes may not be conserved across snail species, and it is likely that immunity constitutes a wide array of variable genes. In *B. glabrata* two such polymorphic loci have been identified and described by Tennessen et al. 2015 and Tennessen et al. 2020 [5, 10]. Named Polymorphic Transmembrane Cluster 1 and 2 (PTC 1 and 2), these regions are each associated with several fold decreased odds of infection. However, the immune allele within PTC 2 likely has higher penetrance than the immune allele within PTC 1, and although the immune allele in PTC 1 is haplosufficient and completely dominant, incomplete dominance is observed for the immune allele within PTC 2. Variation in the genetic mechanisms of immunity can result in altered evolutionary trajectories in the face of the same strength of selection due to infectious miracidia. We show below how variation in dominance and penetrance changes the expected frequency of infection after generations of selection observed in Fig C.

In contrast to naturally-occurring alleles that whose inheritance is governed by the interaction between selection and dominance, an effective gene drive (high homing efficiency) may not be sensitive to this interaction because gene drive heterozygotes are produced at low frequencies, and therefore, dominance plays only a small role in determining the fitness of gene drive alleles. We simulate GDMI inheritance under default conditions to test whether GDMI

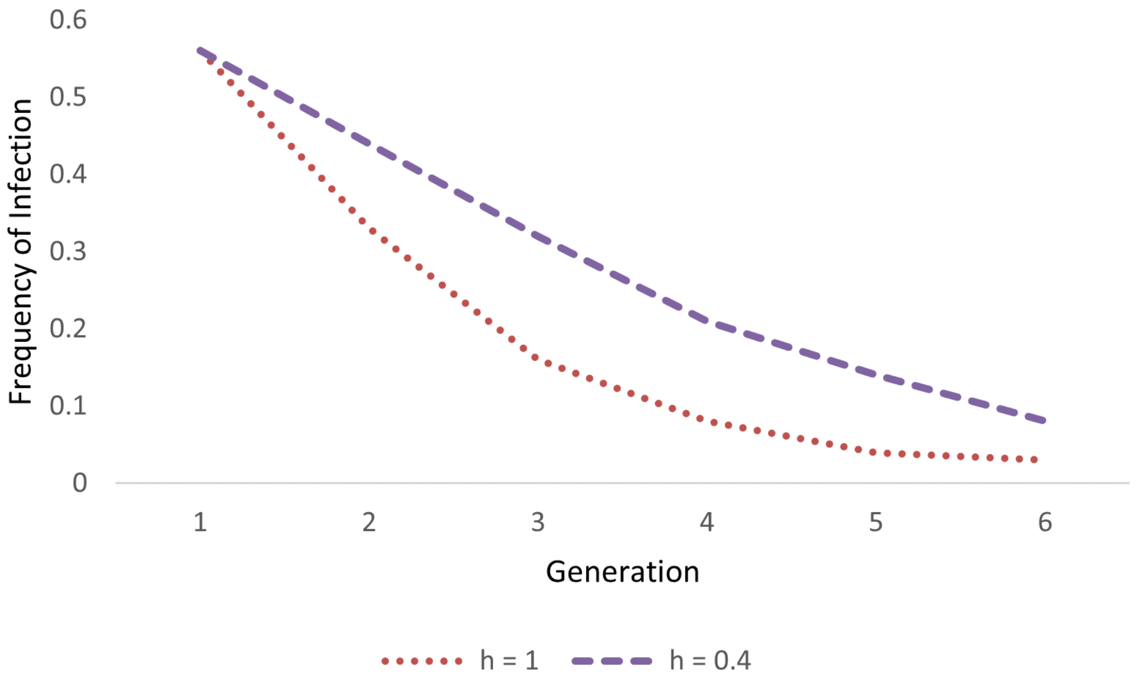


Figure C: The relationship between the immune and susceptible alleles described by the dominance coefficient governs the trajectory of evolution for naturally-occurring immunity. Lower dominance of the immune allele leads to slower evolution of immunity, which could change the speed at which GDMI increases in frequency in a population.

frequency is significantly altered by the strength of dominance of the immune allele over the susceptible allele(s). Dominance coefficients of 1 and 0.4 were chosen as the measured upper and lower bounds for PTC 1 (h = 1) and PTC 2 (h = 0.4). The results in Fig D show that the effect of dominance on the success of gene drive in the 10 year evaluation window is minimal. These results support the notion that an effective drive designed targeting either locus would operate similarly provided other factors are equivalent.

Two caveats may change these results: PTC 2 contains 2 susceptible alleles, therefore a natural heterozygote of both susceptible allele exhibits intermediate immunity compared to natural homozygotes of each susceptible allele, and penetrance of immunity associated with PTC 2 was measured higher than PTC 1 (approx. 2-fold higher odds ratio). In the case of two susceptible alleles displaying a range of immunity across natural susceptible genotypes, independent assortment ensures that relative fitness of immune alleles will depend on the average absolute fitness of the susceptible alleles. The average absolute fitness of susceptible alleles is a byproduct of their interactions to produce a range of susceptible phenotypes. One susceptible allele in PTC 2 is additive: the homozygote is twice as susceptible as the heterozygote (one susceptible allele, one immune allele). The other susceptible allele in PTC 2 is partially additive: the homozygote is less than twice as susceptible as the heterozygote. Barring other epistatic interactions, the measured susceptibility of the genotypes, their frequency, and the force of infection (directional selection) can be used to determine the relative fitness of immunity. However, differences between the alleles, including costs of maintaining each of the susceptible genotypes, is unknown and precludes investigation into differences between the fitness of the susceptible genotypes in the face of selection. This subject will require further empirical investigation to determine whether a spectrum of susceptible alleles may alter the speed of establishment of GDMI for a PTC 2 -like target. Based on results presented in Fig 4 (main text), GDMI establishment is mildly sensitive to standing genetic susceptibility to infection, thus we expect minor differ-

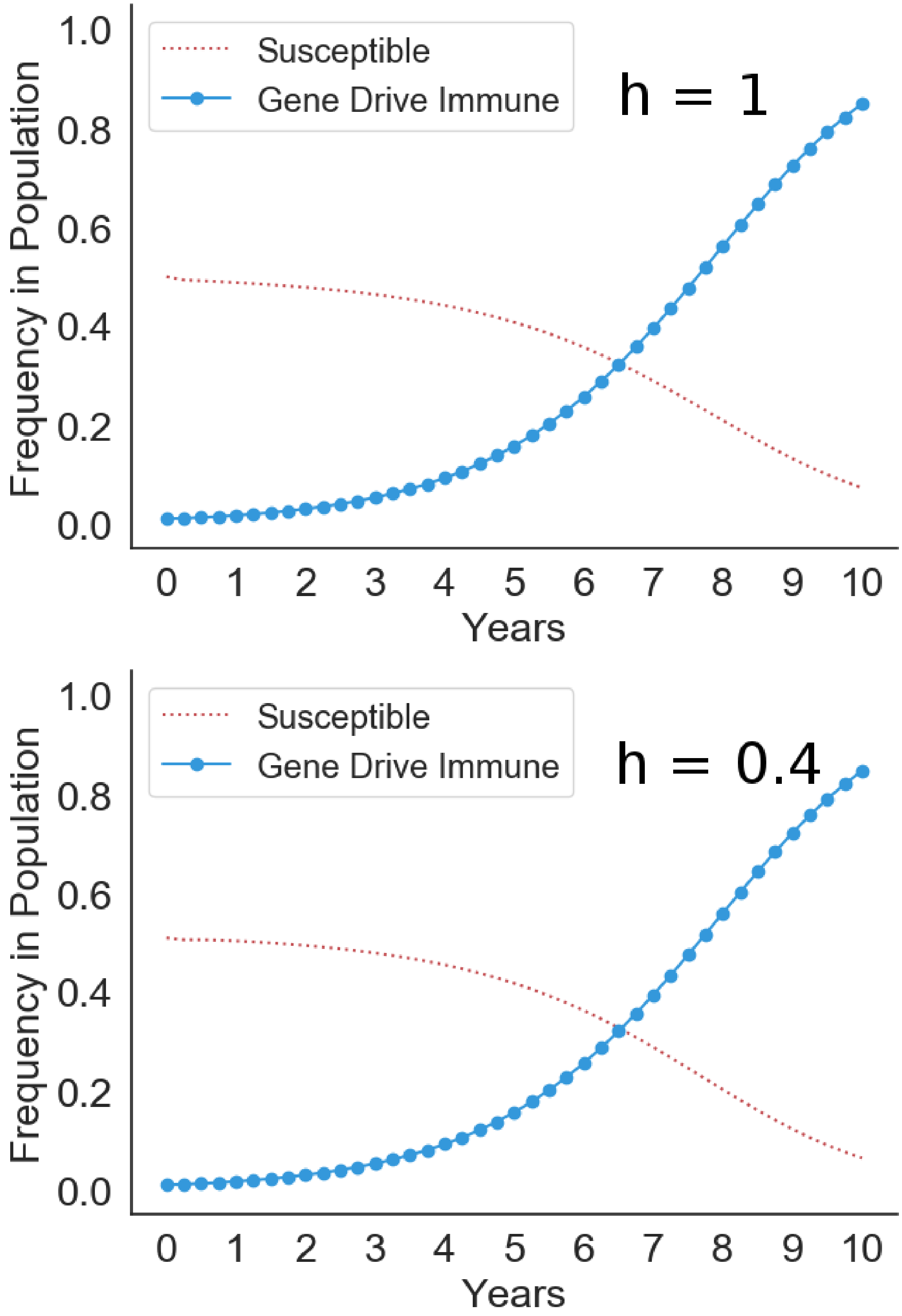


Figure D: High dominance (top panel, h=1) representing PTC 1 and low dominance (bottom panel, h=0.4) representing PTC 2 do not yield measurably different results under default simulation conditions after 10 years.

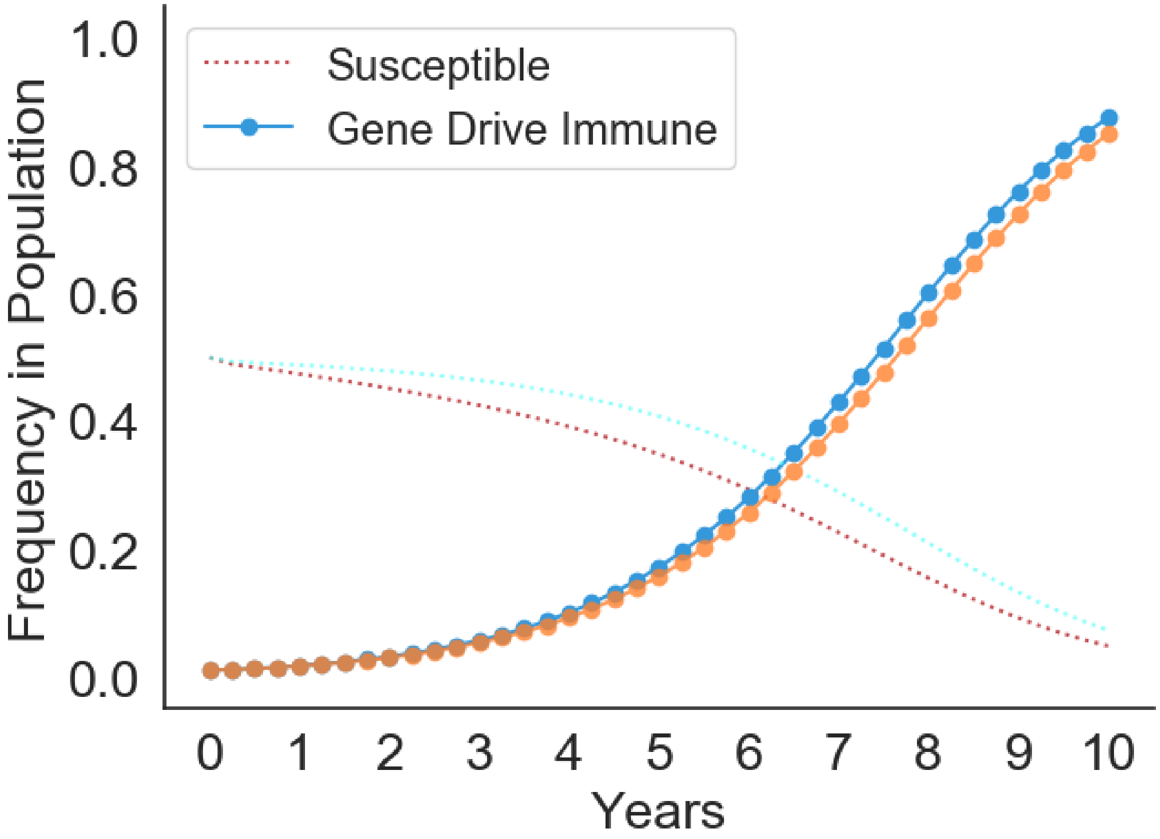


Figure E: The effect of default penetrance ($\iota = 0.8$) compared to higher penetrance ($\iota = 0.9$) in the establishment of GDMI. Higher penetrance produces the blue GDMI and red susceptible lines, while lower penetrance produces the orange GDMI and light blue susceptible lines.

ences in the evolutionary dynamics between a single susceptible allele system and a diversified susceptible allele system. A factor that has greater potential for impact on the evolutionary dynamics of GDMI is penetrance. Higher penetrance of immunity results in greater phenotypic variation with a heritable basis, which provides greater evolutionary potential. Immunity associated with PTC 1 is modeled with $\iota = 0.8$. Susceptibility associated with PTC 2 represents up to 2-fold greater odds of infection. We simulate with $\iota = 0.9$ in Fig E.

Despite the 2-fold greater odds of infection, PTC 2 susceptibility is not a significantly better target for GDMI. Resulting immunity in the population is similar after 10 years. These results

change as the fitness advantages of GDMI over natural immunity diminish (e.g. low homing efficiency) and the fitness advantages of immunity over susceptibility strengthen (e.g. high force of infection).

## Generation time and population turnover

The evolutionary dynamics for GDMI reported here describe conditions in which the average time to reproduction is 3 months and the natural background mortality is half of the adult population in that time. However, when fortuitous environmental conditions prevail, or for snail species with shorter generation times, the establishment of GDMI in a population may happen at a different speed. In ten years, genotype frequencies in the population may be far different for these variable conditions. We demonstrate how variation of two basic life history parameters – natural mortality rate and generation time (mean time to reproduction) – influence the establishment of GDMI in 10 years. Fig F displays simulations under default conditions, while these two life history parameters vary.

## Invasion conditions

Each genotype can be determined to be invading given that it is increasing in frequency at time $t = 0$. Invasion of a genotype does not guarantee increasing frequency at any time $t$, as conditions may change, even in the deterministic model (i.e. model results are not monotonic). However, invasion criteria are important determinants in understanding the behavior of the genetic system in the early stages of gene drive release or even in a natural but unstable genetic system (e.g. strong directional selection). For GDMI establishment, the relative fitness of the

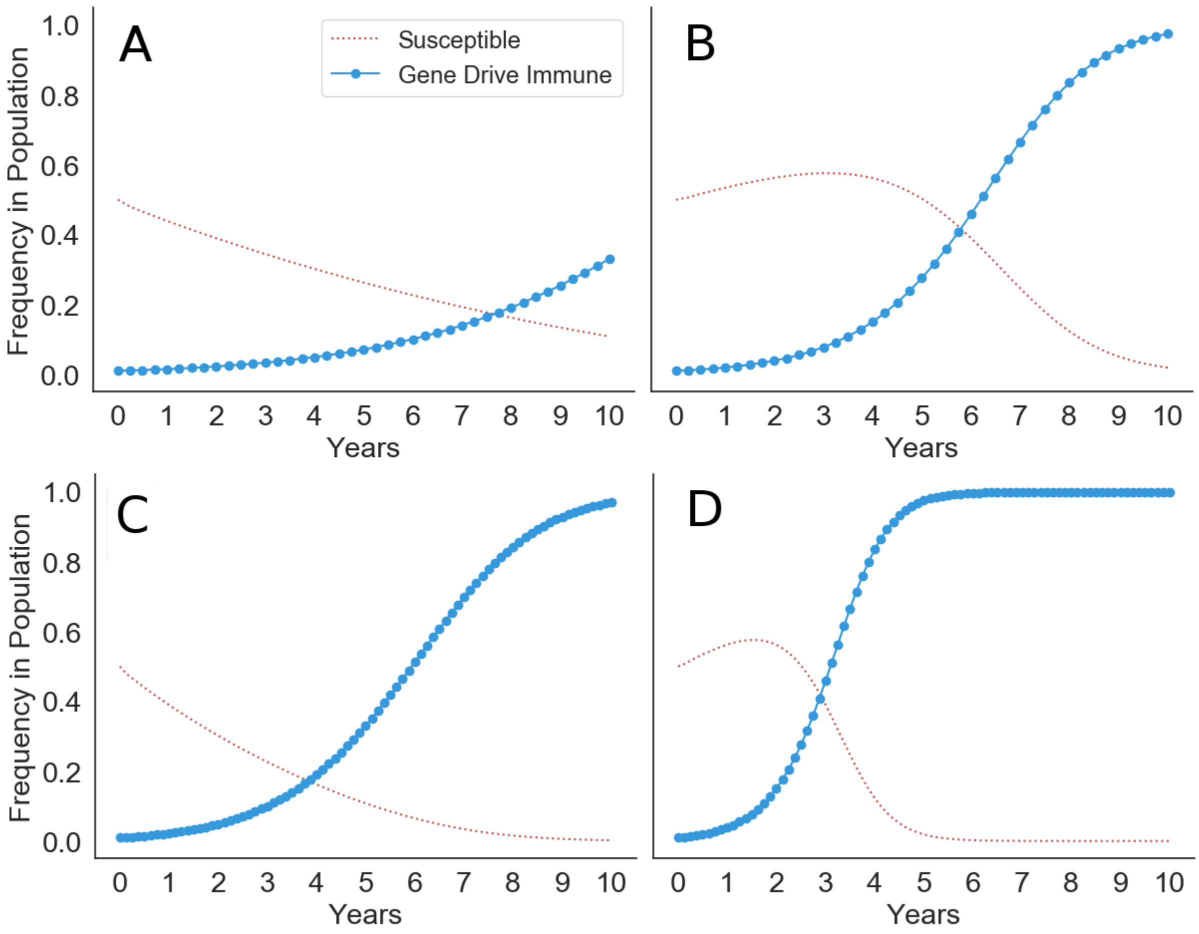


Figure F: Simulations of susceptible and GDMI frequencies under variable life history strategies, namely mean generation time and death rate. Increasing death rate results in more population turnover each generation and more rapid fixation of GDMI. Panel A shows results for $\mu = 0.25$ while panel B shows results for $\mu = 0.75$. Similarly, shorter generations yields more rapid fixation of GDMI in 10 years because more generations occur within the time window. Panels C and D give show results for a mean generation time of 1.5 months (80 generations in 10 years) in contrast to 3 months (40 generations in 10 years). Panel C maintains $\mu = 0.25$, and panel D maintains $\mu = 0.75$.

gene drive homozygote must be greater than 1. This can be directly determined by ensuring:

$$\frac{\lambda_6(0)}{\lambda(0)} > P_{33}(0) \tag{76}$$

$$\implies \frac{\lambda_6(0)}{\lambda(0)P_{33}(0)} > 1 \tag{77}$$

Additionally, the total population size must not decline towards extinction for this invasion to be successful. In Fig G we calculate invasion thresholds for the variety of model parameters in relation to self-fertilization frequency.

Selfing rate and homing efficiency interact to form a curved region bounding values above $H = 0.6$ and below $\sigma = 0.8$. Homing efficiencies below 0.6 do not produce a viable gene drive under default conditions for any selfing rate. Similarly, selfing rates above 0.8 render a gene drive less fit than the natural population and unable to invade. Also supported by Fig 4 (main text), the cost of the payload is a strong determinant in the success of the invasion of GDMI. Perhaps strongest is the influence of gene flow on the invasion of GDMI, which restricts invasion to a small subset of conditions, rapidly excluding snail species with moderate selfing rates as gene flow increases. This does not capture long-term dynamics where GDMI individuals immigrate to the focus population – a process that may occur if GDMI establishes in the neighborhood of the focus population. Highlighted here is the robustness of invasion across the range of disease conditions. In both low and high transmission areas, invasion of GDMI is possible for some or all snail species. Species exhibiting high selfing rates may be invaded by GDMI in high transmission areas.

# Extinction risk

Invasion thresholds are valid and provide context for the conditions in which GDMI will proliferate given that the GDMI allele is not lost from the population due to genetic drift. Drift is strongest in generations immediately proceeding introduction (in a homogeneous environment)

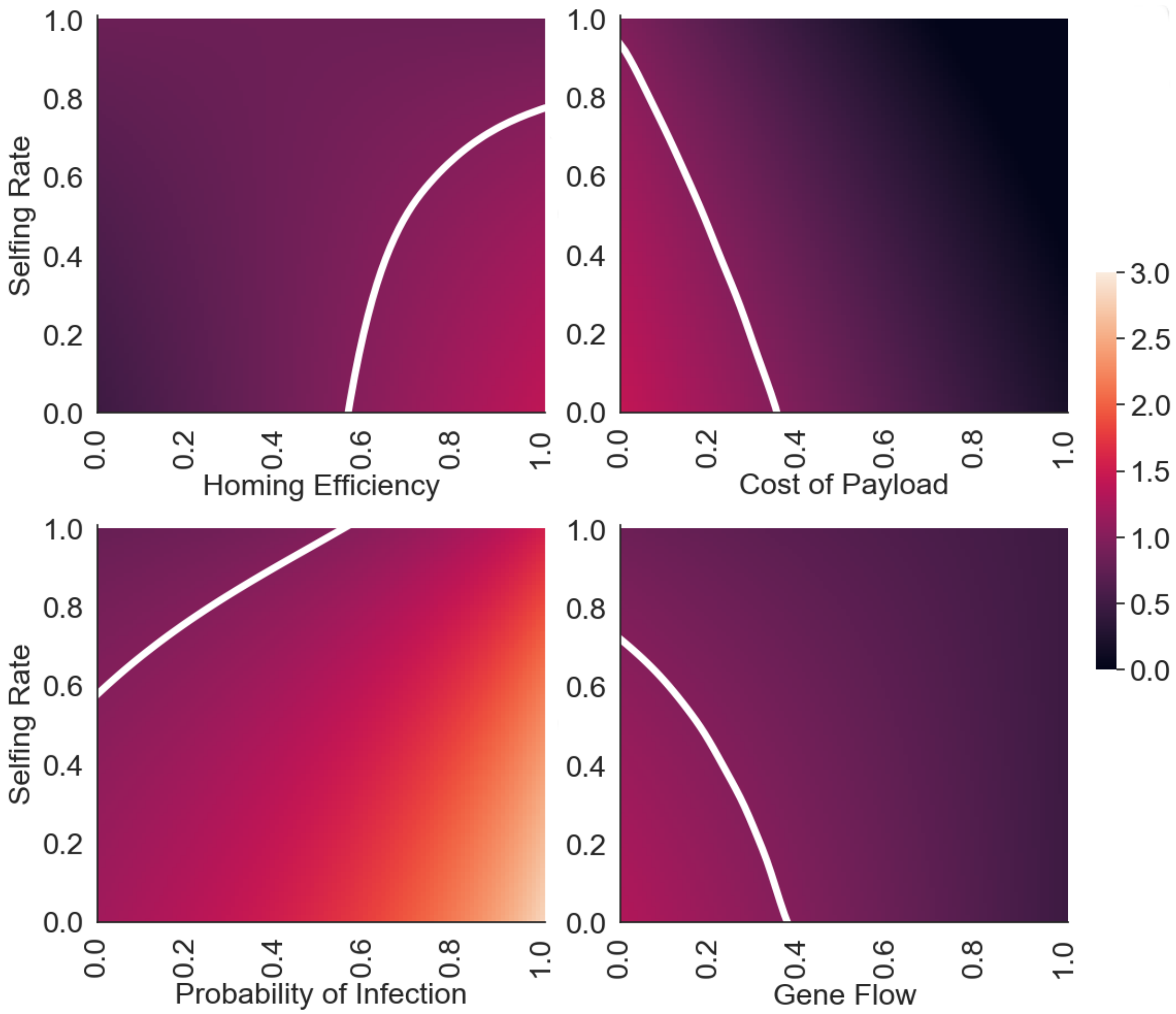


Figure G: Invasion analyses for variables that influence the probability of invasion. Other parameters are held at their default value according to Table A, while the reproduction number is calculated as selfing rate varies. Lighter areas indicate higher reproduction numbers, and white lines represent the isocline at threshold conditions ($R_0 = 1$). The ratio reported in equation 77 and $R_0$ share a value of 1 under threshold conditions but are otherwise not precisely equal due to the nature of overlapping generations in the model.

when the size of the pool of GDMI alleles is small. In contrast to a deterministic invasion process, genetic drift depends on the size of the seed GDMI population. We show how the probability of extinction of GDMI in 10 years (40 generations) varies with the size of the seed population and the absolute fitness of GDMI. We assume the probability of extinction is driven by a stochastic death process contributed through background mortality and infection prior to reproduction in each generation. Default parameters for background mortality and infection for GDMI homozygotes are $0.5\ gen^{-1}$ and $0.03\ gen^{-1}$, respectively. Therefore, we model the stochastic death process as $Pr(X = k\ deaths) = B(n, 0.53)$, where $n$ is the number of GDMI homozygotes (excludes heterozygotes for simplicity due to their transiency at $H = 0.9$). Given a geometric mean absolute fitness $f$ and number of generations from introduction, $t$, the cumulative distribution function representing the probability of extinction at time $t$ is

$$Pr(X \leq (t+1)) = Pr(X \leq t) + 0.53^{nf^{t+1}}(1 - 0.53^{nf^{t}})(1 - Pr(X \leq t)) \tag{78}$$

We calculate the probability of extinction within 40 generations ($t = 40$) using this recursive formulation and plot the results in Fig H across a range of absolute fitness values and number of seeded GDMI individuals in the focal population.

Results indicate that the probability of extinction depends primarily on the absolute fitness of GDMI and little on the number of seeded individuals. Extinction is guaranteed below geometric mean absolute fitness of 0.9, regardless of the size of the introduced GDMI cohort. At low numbers, the threshold of extinction resides at a fitness of 1. This threshold is stark, with very little intermediate extinction risk in 40 generations. This is due primarily to the number of generations simulated; fewer generations would yield more intermediate extinction probabilities on the same plot. Combined with earlier results, this shows that the size of the introduced cohort is important for rapid fixation of GDMI but less important for persistence of GDMI in the population. This conclusion may not hold when reproduction and death is highly variable due

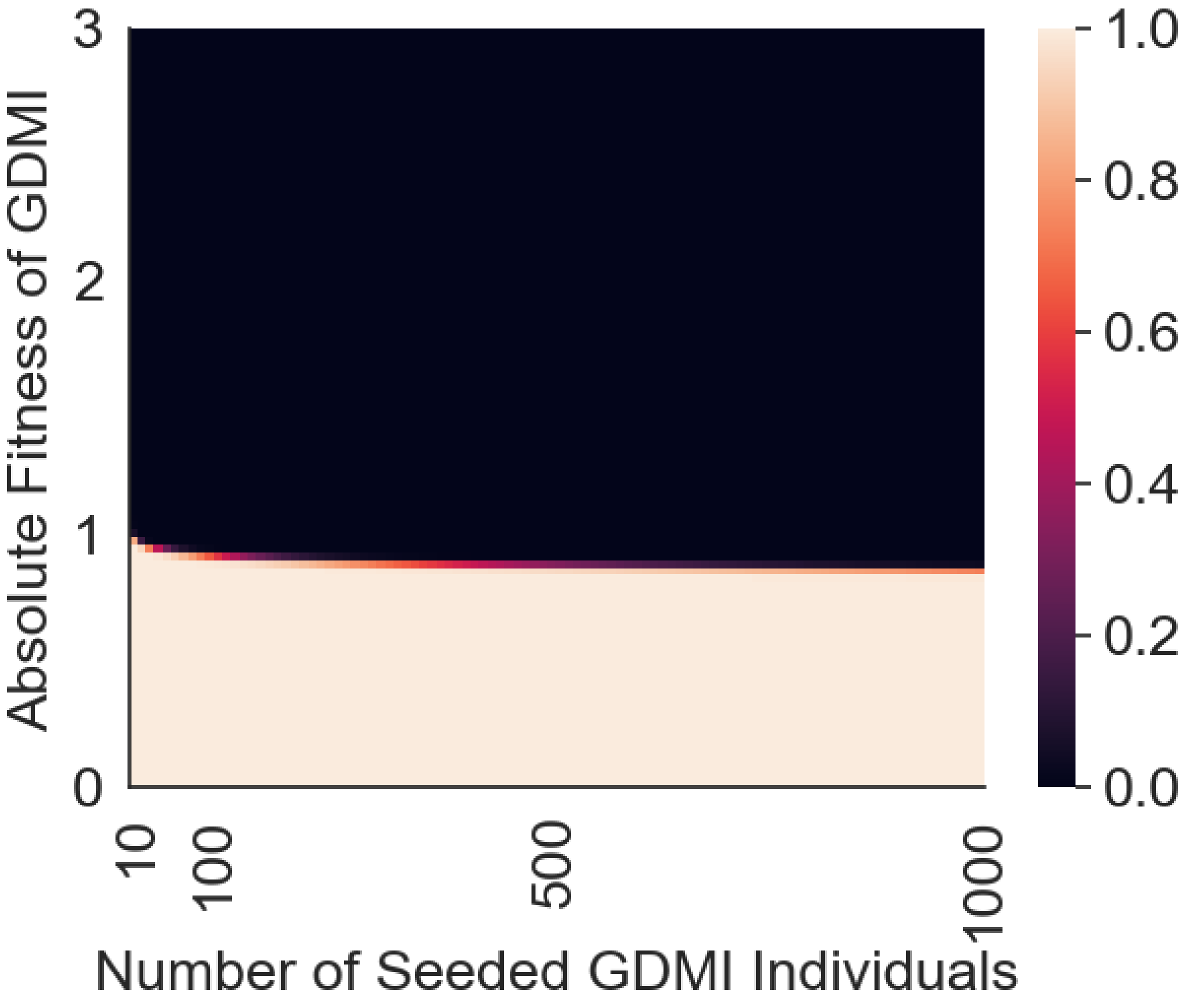


Figure H: The probability of extinction within 40 generations according to absolute fitness and the number of seeded GDMI individuals. Darker values represent low likelihood of extinction.

to factors like seasonality. Higher variability will result in higher extinction risk, particularly for smaller seed populations.

## Seasonality

Dramatic variation in available snail habitat due to seasonal changes in precipitation is common in schistosomiasis endemic regions. Highest variation is observed in sites with ephemeral water bodies and agricultural areas. It is unclear how seasonal variation in habitat availability will alter the speed of establishment of GDMI. We compare GDMI establishment with and without seasonality in carrying capacity of the snail population, with a four fold change in carrying capacity simulated in the seasonally variable population. Fig I demonstrates that seasonality slows the establishment of GDMI, even in a deterministic model. Although not shown in Fig I, higher variability in carrying capacity corresponds monotonically to slower establishment of GDMI. These results support the use of GDMI in sites with less seasonal variability in snail population abundance.

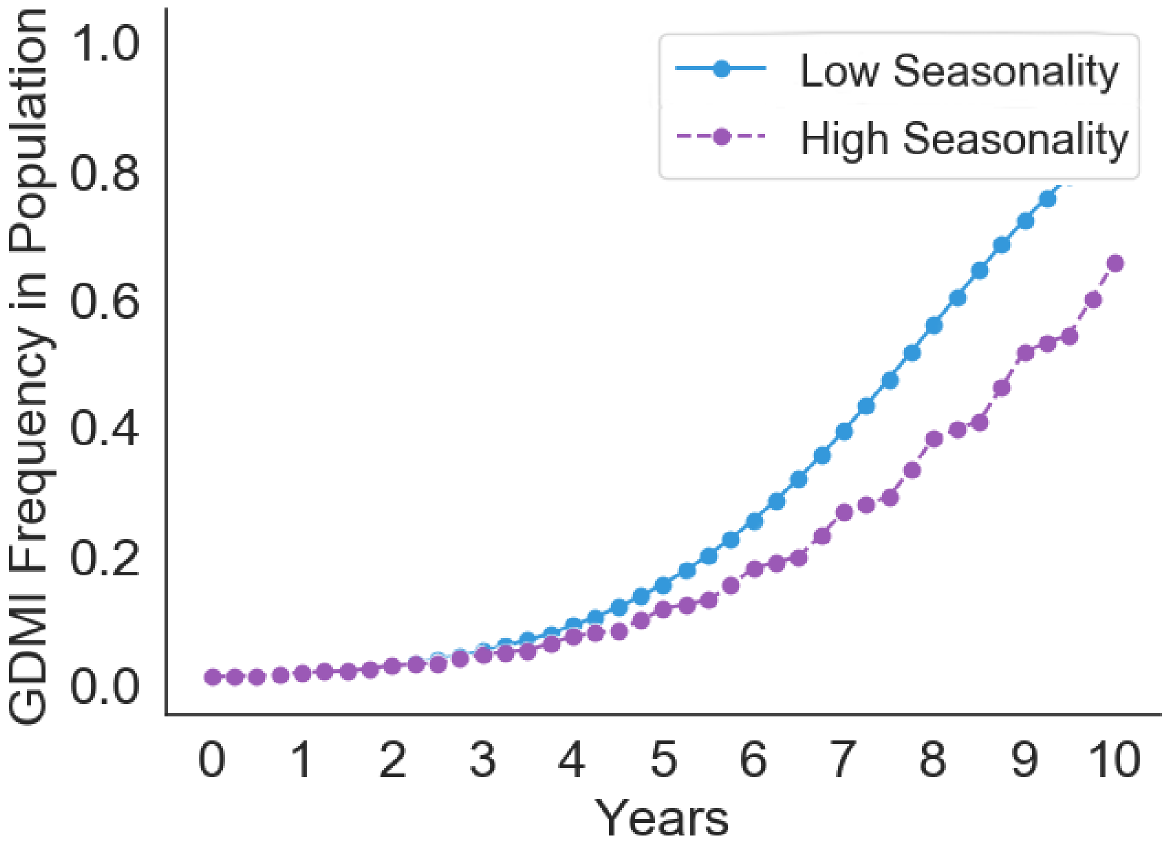


Figure I: The spread of GDMI in a population with fluctuating carrying capacity due to seasonal rainfall and habitat variation. High seasonality assumes at 4 fold change in carrying capacity in 2 generations, with a full cycle occurring in 4 generations (equal to 1 year with default generation time): 200 %, 100 %, 50 %, 100% carrying capacity cycle. Low seasonality assumes no fluctuation in carrying capacity.

# Epidemiological model

Here we modify the classic MacDonald model for schistosome transmission to include the frequency of resistant snails that occurs in the environment due to introduction and subsequent spread of GDMI in the population. The simplest form this takes is to subtract the frequency of immune snails from the susceptible snail frequency such that the frequency of susceptible snails is given by $(1 - y - \rho)$, where $\rho$ is the frequency of immunity (natural and engineered). The resulting system of coupled ordinary differential equations is given below (equations 5 and 6):

$$\frac{dw}{dt} = \alpha y - \mu w \tag{79}$$

$$\frac{dy}{dt} = \Lambda^*(1 - e^{-\beta w})(1 - y - \rho) - vy \tag{80}$$

These two equations govern the prevalence of infection in snails, $y$, and the mean per capita worm burden in humans, $w$. The distribution of adult worms in the human population is assumed to approximate a negative binomial distribution. Parameters and their values are described in Table B.

We evaluate the efficacy of GDMI intervention by comparing mean worm burden after a ten year period with the mean worm burden at equilibrium endemic conditions. We calculate transmission rates $\Lambda$ and $\alpha$ at endemic equilibrium. Let $w^*, y^*$ be nontrivial equilibria for which $\frac{dw}{dt} = \frac{dy}{dt} = 0$.

$$w^* = \frac{\alpha y}{\mu} \tag{81}$$

$$y^* = \frac{(1 - \rho)\Lambda(1 - e^{-\beta w})}{\Lambda(1 - e^{-\beta w}) + v} \tag{82}$$

$y^*$ is frequently estimated in field surveys and may vary across sites or through the year due to seasonal variability in rainfall and human use of aquatic snail habitat like drainage areas,

Table B: Parameter values for the epidemiological model

| Parameter | Description | Value | Ref. |
|---|---|---|---|
| $\alpha$ | transmission rate converting snail infections to adult worms in humans | $142\ wk^{-1}$ | calculated based on model equilibrium at $w^* = 710$, $y^* = 0.02$ |
| $\mu$ | death rate of adult worms | $0.004\ wk^{-1}$ | Harmonic mean of range of $(3 - 7yrs)^{-1}$ commonly reported in literature across *Schistosoma spp.* [11] |
| $\Lambda^*$ | force of infection from humans to snails at endemic equilibrium | $0.0104\ wk^{-1}$ | derived from epidemiological model |
| $\rho$ | fraction of immune snails | variable, $\rho^* = 0.5$ | determined by genetic model |
| $v$ | death rate of infected snails | $0.25\ wk^{-1}$ | Harmonic mean of death rates of infected *Bulinus globosus* and *Biomphalaria pfeifferi* [3] |
| $b$ | per capita worm to snail transmission rate | $3 * 10^{-5} w^{-1}$ | calculated based on model equilibrium at $w^* = 710, y^* = 0.02$ |
| $\beta$ | human population to snail transmission rate | variable, $\beta^* = 0.0147\ wk^{-1}$ | calculated: $\beta = \frac{1}{2}\phi b$ |
| $m$ | per capita mean number of mated pairs of adult worms | variable, $m^* = 348$ | calculated: $m = \frac{1}{2}\phi w$ |
| $k$ | clumping parameter: $NB(w, k)$ | 0.24 | fitted to *S. mansoni* data [12] |
| $w$ | per capita mean worm burden | variable, $w^* = 710$ | calculated based on model equilibrium at $\Omega = 0.80$ |
| $y$ | frequency of patent infections in snail population | variable, $y^* = 0.02$ | field-observed average in endemic regions [13] |
| $\phi$ | per adult worm mating probability | variable, $\phi^* = 0.98$ | calculation based on $NB(w, k)$ |
| $\Omega$ | per capita prevalence of at least one mated pair of adult worms | variable, $\Omega^* = 0.80$ | field-observed average in endemic and hyperendemic regions [14, 15] |

irrigation ditches, or natural water bodies. Despite variation, low infection prevalence (0-5%) in snails is observed, even in hyperendemic areas. Explanations for low prevalence are multifactorial and relate to the duration of patency, increased mortality rate of patent snails, heterogeneous exposure to miracidial infection, partially evolved immunity to infection, and competition with other trematodes. $w^*$ is not as easily estimated, as measurements rely on quantification of shed eggs in urine and fecal samples. The quantity of eggs is correlated but not linearly related to the number of paired worms, as human immunity leading to granulomatous formation around released eggs as well as potential interactions among adult worms and variability in egg production can obscure the relationship between eggs shed and worm burden. Human autopsies performed on known and suspected schistosomiasis cases reveal differential distribution of eggs and associated pathology with increasing intensity of infestation. Cheever (1968) observed that fewer eggs were present in the rectal mucosa and feces of *S. mansoni* infected individuals with associated fibrosis of the liver. This demonstrates that pathology, intensity, and egg count are not directly related, and the nature of their relationship requires biological knowledge of both the distribution of worms across tissue and the interactions between worms and the immune system. Despite these limitations, a reasonable heuristic is a 1:1 ratio of adult worm mated pairs and eggs per gram (EPG) in feces (*S. mansoni*). Multiple lines of evidence, including challenge experiments in mice, organ specific autopsies and perfusions, as well as observed distributions of EPG in human populations suggests that per capita mean worm burden (MWB) in highly endemic areas can exceed 1000. We simulate moderate-high endemicity with an infection prevalence of $\Omega^* = 0.80$. When the prevalence of infection is $<< 1$, a proportion of adult worms fail to pair with a mate and reproduce. The number of mated pairs can be calculated given the MWB and the distribution of adult worms in the human population. A negative binomial distribution is found to best represent the distribution of adult worms in humans. It is overdispersed, and dispersion increases as prevalence decreases. Prevalence of detectable eggs,

and therefore successfully mated pairs is given as

$$\Omega(w,k) = 1 - 2(1+\frac{w}{2k})^{-k} + (1+\frac{w}{k})^{-k} \tag{83}$$

and $w$ is calculated via substitution of the known prevalence of infection, $\Omega$, and aggregation parameter, $k$, and solving numerically. An equal ratio of male and female schistosomes is assumed in this calculation, as is that the rates of transmission between the two sexes are equivalent [16]. We also assume that both sexes transmit together, and there is no sex-specific compartmentalization in the human body that would limit pairing of adult schistosomes. $w^* = 710$ occurs at an endemic equilibrium prevalence, $\Omega$, of $80\%$. Given $w^* = 710$ and $y = 0.02$, $\alpha$ can be calculated as

$$\alpha = \frac{w^*\mu}{y^*} = 142 \tag{84}$$

In contrast the $\alpha$, which holds a constant value, $\beta$ is a function of the distribution of worms in the local human population. The distribution changes non-linearly with worm burden and prevalence. For simplicity, we assume that $k$ is invariant as worm burden and prevalence change, although evidence suggests higher aggregation with higher burden in some populations. $\beta$ takes the form:

$$\beta = \frac{1}{2}b\phi \tag{85}$$

in which $b$ is a transmission constant that relates the per capita number of mated worm pairs, $m$, to new infections in snails.

$$m = \frac{1}{2}\phi w \tag{86}$$

$\phi$ is the mating probability given by the negative binomial distribution where $\delta = \frac{w}{w+k}$.

$$\phi = 1 - \frac{(1-\delta)^{1+k}}{2\pi} \int_0^{2\pi} \frac{(1-cos\theta)d\theta}{(1+\delta cos\theta)^{1+k}} \tag{87}$$

Given $w^*$ and $y^*$, $\Lambda^*$ can be calculated by approximating that $(1 - e^{-\beta w}) \approx 1$ at endemic equilibrium conditions. Equation 80 simplifies to:

$$\frac{dy}{dt} = \Lambda^*(1 - y - \rho) - vy \tag{88}$$

Solving for the nontrivial equilibrium yields:

$$\Lambda^* = \frac{vy^*}{1 - y^* - \rho^*} = 0.0104 \tag{89}$$

The probability of infection per generation, $Pr(y^+)$, can be approximated from the force of infection and the differential equation for snail infection prevalence:

$$Pr(y^+) \approx \int_{t=\tau}^{\tau+1} \Lambda^*(1 - e^{-\beta w})dt \tag{90}$$

This expression represents the per capita number of snail infections expected in a susceptible population in a generation ($t = \tau$ weeks).

We do not yet have an estimate for $\beta$ (variable) and therefore no estimate for $b$ (constant). These values we determine by calibrating the model with known values for $R_0$ in moderate-high transmission sites. The magnitude of $R_0$ has never been precisely measured for schistosomiasis, as doing so would require measurements of innate immunity in the snail population. It is unknown whether genetic immunity provides cross protection for other trematode species, and therefore, even in a previously schistosome-naive area, pre-existing immunity requires measurement through challenge experiments. With this caveat in mind, $R_0$ measurements are widely thought to exist in the range of 2-5 for schistosomiasis, likely exceeding 3 in moderate-high transmission sites [17]. In a fully susceptible snail population, these values will be higher, and

in all likelihood empirically measured $R_0$ values underestimate true $R_0$ values predicated on a fully susceptible host population. From our system of differential equations we calculate the effective reproductive number $R_t$ and from it, derive the $R_0$ under conditions of partial immunity in snails to calibrate $\beta$. Linearizing the system of equations with respect to $w$ and $y$, we form the transmission and transition matrices outlined by Diekmann et al. in their next generation matrix (NGM) approach to calculate $R_0$ [18]. We extend this approach by relaxing the assumption that the populations of snails and humans are fully susceptible and that no disease is present before an index case. Doing so, we calculate transmission and transition matrices for $R_t$ as:

$$\mathbf{T_t} = \begin{pmatrix} 0 & \alpha \\ (1-\rho)\beta\Lambda^* e^{-\beta w} & 0 \end{pmatrix} \tag{91}$$

$$\mathbf{\Sigma_t} = \begin{pmatrix} -\mu & 0 \\ -y\beta\Lambda^* e^{-\beta w} & -(v + \Lambda^*(1+e^{-\beta w})) \end{pmatrix} \tag{92}$$

We calculate the time-varying NGM as:

$$\mathbf{K_t} = -\mathbf{T_t}\mathbf{\Sigma_t^{-1}} = \begin{pmatrix} \frac{-\alpha\beta y\Lambda^* e^{-\beta w}}{\mu(\Lambda^*(1+e^{-\beta w})+v)} & \frac{\alpha}{\Lambda^*(1+e^{-\beta w})+v} \\ \frac{(1-\rho)\beta\Lambda^* e^{-\beta w}}{\mu} & 0 \end{pmatrix} \tag{93}$$

The expression for $R_t$, computed as the spectral radius of $\mathbf{K_t}$, is

$$R_t = \frac{\sqrt{\alpha\beta\Lambda^*(\alpha\beta\Lambda^* y^2 + 4\mu(1-\rho)(\Lambda^* + (\Lambda^* + v)e^{\beta w}))} - \alpha\beta\Lambda^* y}{2\mu(\Lambda^* + (\Lambda^* + v)e^{\beta w}} \tag{94}$$

A derivation of $R_0$ would require setting $\rho = w = y = 0$. However, because empirical measurements of $R_0$ have not accounted for variations in $\rho$, we calibrate $\beta$ from an empirical form of this equation. Specifically, we set $\rho = 0.5$ according to default conditions that are based on empirical measurements of innate immunity in field captured snails. Setting $w = y = 0$ yields the following expression for an empirical $R_0$

$$R_0 = \sqrt{\frac{\alpha\beta\Lambda^*(1-\rho)}{\mu(2\Lambda^* + v)}} \tag{95}$$

and when $\rho = 0.5$, the expression becomes:

$$R_0 = \sqrt{\frac{\alpha\beta\Lambda^*}{2\mu(2\Lambda^* + v)}} \tag{96}$$

Solving for $\beta$ yields

$$\beta = \frac{2\mu R_0^2(2\Lambda^* + v)}{\alpha\Lambda^*} \tag{97}$$

Recall that $\beta$ is a function of the negative binomial distribution of worms, which determines the probability of mating success among adult worms. However, this theoretical construct breaks down for the low numbers assumed in an index case. For at low numbers, $\phi$ would approximate zero for a stationary $k = 0.24$, and extinction is predicted. The concept of $R_0$ would be irrelevant for schistosomiasis if these theoretical predictions were valid. Instead we assume that early transmission of cercariae are highly clustered and that $\phi$ remains high for the purposes of estimating the constant $b$ which scales $\beta$. Setting $\phi = 1$, we achieve

$$b = \frac{4\mu R_0^2(2\Lambda^* + v)}{\alpha\Lambda^*} \tag{98}$$

We set $b$ to a value with one significant digit so that $R_0$ is approximately the median of empirically measured values. This gives $b = 0.03$ and $R_0 = 3.2$. In practical terms, $b$ represents the 'rebound speed', which is the pace infections can accrue after chemotherapy treatment. Under certain conditions, our estimate may represent the low end of this rebound speed due to the assumption of 100% mating success in index case infections. Moreover, our estimate of $R_0 = 3.2$ is calibrated based on prior empirical measurements, which almost certainly underestimate the true $R_0$ as specified by a fully susceptible host population. We do not explicitly

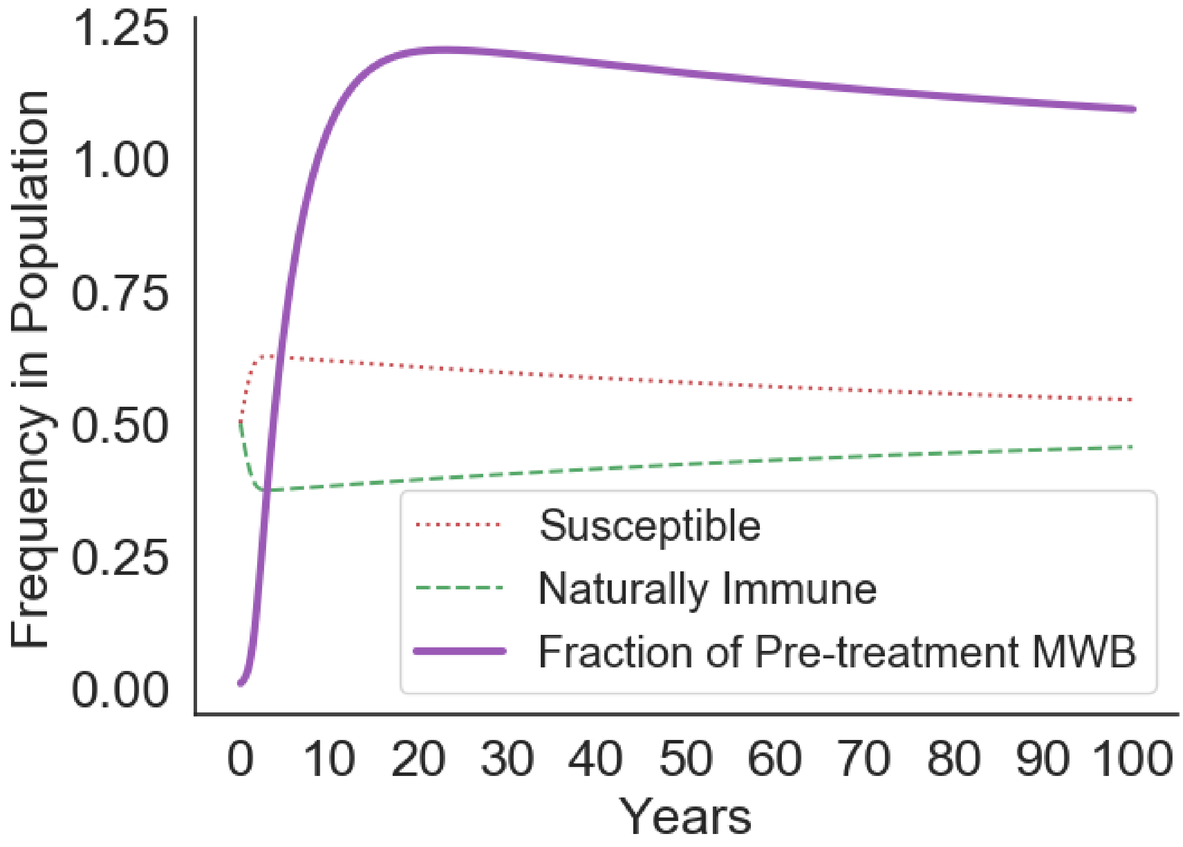


Figure J: Simulation of the emergence of a schistosomiasis epidemic under default conditions. GDMI is not present, and long-term behavior of the model is observed to overshoot endemic equilibrium conditions and return to equilibrium over the course of many years. Susceptibility in snails is advantageous at low levels of infection early in the epidemic and is disadvantageous above equilibrium conditions.

account for adaptive immunity in humans, which has been shown to increase over 10+ years into adulthood. Accounting for evolved innate immunity in snails by setting $\rho = 0$, we find that $R_0 = 4.5$.

In Fig J we show the long-term behavior of the default model without introduction of GDMI. A reproduction number of 3.2 produces a rapid rise of an epidemic past the endemic equilibrium, and as the snail population evolves immunity, an equilibrium is established. Feedback from schistosome transmission produces stabilizing selection on immunity in snails.

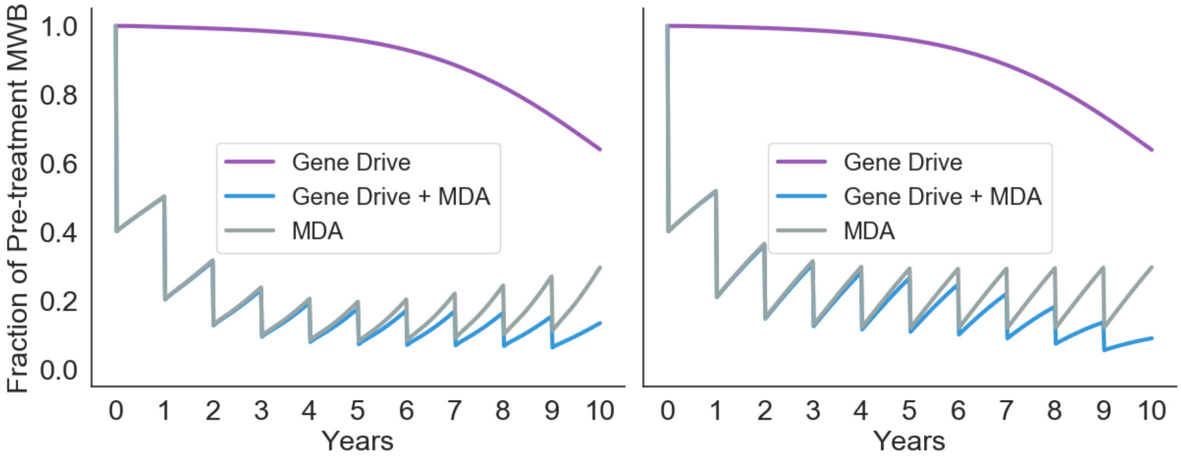


Figure K: Comparative results among three treatment regimes under high and low transmission conditions. $b$ is half of default conditions (left) and $R_0 = 2.3$, producing slower rebounds after annual MDA treatment. More rapid rebounds are observed when $b$ is twice default conditions and $R_0 = 4.5$ (right).

In Fig 5 (main text), GDMI was evaluated in comparison to and with coincident annual MDA treatment. 60% reduction in MWB in the population was modeled for each treatment and is a product of coverage and efficacy of the chemotherapy. Although alone GDMI is not capable of eliminating schistomiasis locally within a 10 year evaluation period, it was shown to successfully complement MDA under simulated conditions to produce greater and more sustained reduction than MDA alone. However, these results may be sensitive to several factors, especially the force of infection to humans which determines how rapidly the human population becomes infected from an infected snail population. Rapid reinfection results in a faster rebound to pre-treatment MWB, and therefore, subsequent treatment is less effective because MWB reduction is not long lasting. We explore high and low transmission conditions by manipulating $b$, which in turn, changes $\beta$. Fig K shows the difference between $R_0 = 2.3$ and $R_0 = 4.5$ conditions as MDA and GDMI are applied.

GDMI performs favorably in absolute reduction in joint use with MDA when transmission is higher, indicating that the efficacy of GDMI is enhanced in conditions that are challenging

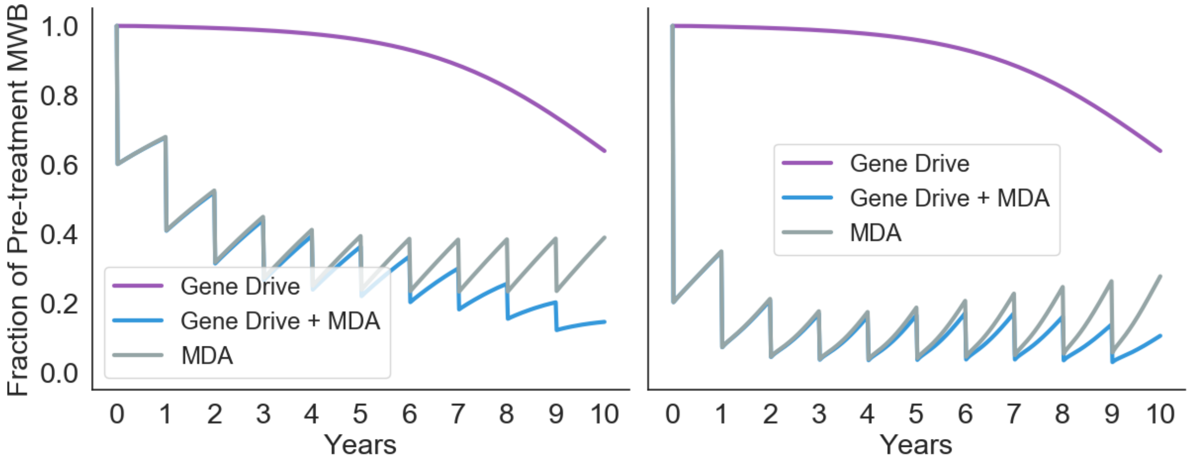


Figure L: Comparative results among three treatment regimes under high and low intensity MDA application in the human population. 40% annual reduction in MWB (left) produces slower elimination across all treatment regimes compared to 80% annual reduction (right). Rebounds are concave down and relatively smaller for lower intensity MDA and concave up for high intensity MDA. This reflects slower loss of immunity, and for joint treatment the faster gain of GDMI, in the snail population due to higher selection pressure in favor of immunity in higher transmission conditions.

for reduction through MDA alone. Additionally, the intensity of MDA treatment may have a strong effect on the benefits of GDMI, as selection pressures are changed. Fig L displays the difference in reduction of MWB between low and high intensity MDA use under equivalent GDMI application.

These results demonstrate diminishing returns for the application of MDA at higher concentrations as immunity in snails evolves to favor higher transmission conditions when adult worms are eliminated quickly. Success of GDMI is slowed when force of infection on snails, and therefore positive selection on immunity, is reduced.

The treatment window of 10 years is common for evaluating funded public health campaigns, though results of this study will differ using longer treatment windows. We extend this window to 40 years to demonstrate the long-term effects of each of the treatment regimes. Additionally, we show that when MDA is remitted after 10 years, GDMI is able to maintain re-

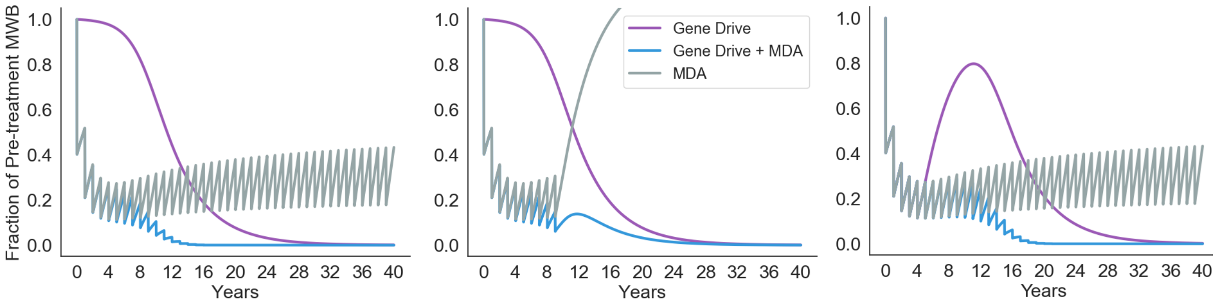


Figure M: Simulations of the three treatment regimes for 40 years. Regimes are continued annually for the duration of the simulation (left). MDA is stopped after 10 years of treatment (middle). GDMI is added five years after existing MDA treatment (right).

ductions in MWB, while without GDMI MWB returns to endemic equilibrium conditions (after an overshoot also depicted in Fig J). In a human population previously treated with MDA to which GDMI is later added, similar patterns to a previously untreated population emerge but on different timelines. With MDA alone, MWB stablizes to a lower level than without treatment. Adding GDMI to this treatment regime leads to long-term elimination. Elimination is achieved with GDMI alone, but in the short-term after MDA is discontinued, MWB increases for a decade before falling again. Elimination is contingent on the continued success of GDMI over decades, which could be challenged by the evolution of resistance to GDMI or extinction, as discussed in the main text and supplement.

# References


1. Webster J and Woolhouse M. Cost of resistance: relationship between reduced fertility and increased resistance in a snail—schistosome host—parasite system. Proceedings of the Royal Society of London. Series B: Biological Sciences 1999;266:391–6.
2. Doums C, Viard F, Pernot AF, Delay B, and Jarne P. Inbreeding depression, neutral polymorphism, and copulatory behavior in freshwater snails: a self-fertilization syndrome. Evolution 1996;50:1908–18.
3. Woolhouse M. The effect of schistosome infection on the mortality rates of Bulinus globosus and Biomphalaria pfeifferi. Annals of Tropical Medicine & Parasitology 1989;83:137–41.
4. Woolhouse M. Population biology of the freshwater snail Biomphalaria pfeifferi in the Zimbabwe highveld. Journal of Applied Ecology 1992:687–94.
5. Tennessen JA, Théron A, Marine M, Yeh JY, Rognon A, and Blouin MS. Hyperdiverse gene cluster in snail host conveys resistance to human schistosome parasites. PLoS genetics 2015;11:e1005067.
6. Unckless RL, Clark AG, and Messer PW. Evolution of resistance against CRISPR/Cas9 gene drive. Genetics 2017;205:827–41.
7. Escobar JS, Auld JR, Correa AC, et al. Patterns of mating-system evolution in hermaphroditic animals: Correlations among selfing rate, inbreeding depression, and the timing of reproduction. Evolution: International Journal of Organic Evolution 2011;65:1233–53.
8. Gantz VM and Bier E. The mutagenic chain reaction: a method for converting heterozygous to homozygous mutations. Science 2015;348:442–4.
9. Karlin S. Equilibrium behavior of population genetic models with non-random mating. Part I: Preliminaries and special mating systems. Journal of Applied Probability 1968;5:231–313.
10. Tennessen JA, Bollmann SR, Peremyslova E, et al. Clusters of polymorphic transmembrane genes control resistance to schistosomes in snail vectors. Elife 2020;9:e59395.
11. Goddard M and Jordan P. On the longevity of Schistosoma mansoni in man on St. Lucia, West Indies. Transactions of the Royal Society of Tropical Medicine and Hygiene 1980;74:185–91.
12. Mangal TD, Paterson S, and Fenton A. Predicting the impact of long-term temperature changes on the epidemiology and control of schistosomiasis: a mechanistic model. PLoS one 2008;3:e1438.
13. Anderson R and May R. Prevalence of schistosome infections within molluscan populations: observed patterns and theoretical predictions. Parasitology 1979;79:63–94.

14. Chan M, Guyatt H, Bundy D, Booth M, Fulford A, and Medley G. The development of an age structured model for schistosomiasis transmission dynamics and control and its validation for Schistosoma mansoni. Epidemiology & Infection 1995;115:325–44.

15. Tchuenté LAT, Momo SC, Stothard JR, and Rollinson D. Efficacy of praziquantel and reinfection patterns in single and mixed infection foci for intestinal and urogenital schistosomiasis in Cameroon. Acta tropica 2013;128:275–83.

16. May RM. Togetherness among schistosomes: its effects on the dynamics of the infection. Mathematical biosciences 1977;35:301–43.

17. Woolhouse M, Hasibeder G, and Chandiwana S. On estimating the basic reproduction number for Schistosoma haematobium. Tropical Medicine & International Health 1996;1:456–63.

18. Diekmann O, Heesterbeek J, and Roberts MG. The construction of next-generation matrices for compartmental epidemic models. Journal of the Royal Society Interface 2010;7:873–85.
