# Supplementary material for: Modeling the efficacy of CRISPR gene drive for snail immunity on schistosomiasis control
Source: PLoS Negl Trop Dis. 2022 Oct 31;16(10):e0010894. doi: 10.1371/journal.pntd.0010894 (PMC9648845; doi:10.1371/journal.pntd.0010894)
Supplement: Attachment — Submitted filename: ResponseLetter_PLosNTD.pdf [file pntd.0010894.s002.pdf]

Stanford University

*Hopkins Marine Station*
*120 Oceanview Blvd*
*Pacific Grove, CA 93950*

RE: Response to reviews of "Modeling the efficacy of CRISPR gene drive for schistosomiasis control" in *PLoS NTD*

Dear editors Dr. Rinaldi, Prof. Geary, and reviewers Dr. Oettle and Dr. Crellen,

Thank you for your time in assessing our manuscript PNTD-D-22-00821, "Modeling the efficacy of CRISPR gene drive for schistosomiasis control." We are grateful for the constructive reviews and the time you spent improving our manuscript. We have carefully considered all of your comments and revised the manuscript and further explained accordingly. We have organized our revisions by reviewer below.

Reviewer 1: Dr. Oettle

- We agree that because some literature indicates that high exposure can accelerate acquisition of immunity, especially in children, it is more accurate to rephrase as suggested. We have re-written the sentence in the caption of figure 1 accordingly: "Existing literature does not consistently support the role of variable human immunity in epidemiological models in endemic regions, therefore immunity in the human population is assumed constant."
- We have clarified the location of parameter estimates and sensitivity analyses. In line 160 (Results section), we have added references to "figures 3 and 4" for sensitivity analyses of parameters that are treated as variables to explore a range of simulated conditions. All parameter estimates are provided in tables S1 and S2 with reference to their method of derivation from the literature or analytically from the model. When values were derived analytically, the calculations are described in the supplemental equations. The sensitivity analyses in figures 3 and 4 of the main text were produced by substituting the indicated range of values for the parameter represented by each panel with bootstrapping conducted for a range of inbreeding fitness costs (fig. 3) and then selfing rates (fig. 4) to yield confidence intervals. We have clarified in the caption of fig. 3 that bootstrapping was conducted to produce the confidence intervals.
- We have included an additional sentence in lines 278-281 (Discussion section) to relate snail species to the potential schistosome species treated with GDMI: "*Schistosoma japonicum* may be treated most effectively with GDMI because *O. hupensis* is not known to self-fertilize, whereas *S. mansoni* and *S. haematobium* are transmitted by snail species with high and low selfing rates, making ecological surveys and feasibility modeling crucial in advance of intervention for these parasite species." Also on lines 303-305 we have included discussion on the relationship between selfing of snails and isolation of habitat: "Because selfing is encouraged for isolated snail populations, a trade-off between selfing rate and size of an interbreeding snail population may prove challenging to optimize GDMI introduction." Because several species of snails that host schistosomes have not been investigated for selfing rates and because the species on which we have information live in diverse habitats, this is the strongest comment we can make about the relationship between selfing rates in snail species and habitat connectivity.
- All notation errors were corrected and highlighted. References and labels in the latex document could not be highlighted with the package used, so the word(s) next to these

corrections were highlighted when necessary to indicate their placement.

Reviewer 2: Dr. Crellen

- We agree that the title could be more specific to the type of gene drive proposed and have amended the title to: "Modeling the efficacy of CRISPR gene drive for snail immunity on schistosomiasis control". We also have added to the discussion in lines 292-294: "Although CRISPR represents the most likely technology for developing GDMI constructs, it may not be the only successful gene editing method; our results cover a wide range of scenarios that also apply to non-CRISPR drives."
- "several forms of cancer" was not specific, and we have corrected the language to reflect the cancers that are associated with schistosomiasis: "bladder and colorectal".
- We have condensed equations 8 and 9 in the supplement to provide a simplified model of population replacement in equation 5 of the main text as well as additional text to explain the equation. "A simplified model of death, migration, and recruitment is given below in equation 5: [equation 5] Each genotype is indexed by $i$, $N$ represents the population size, $\gamma$ is the finite death rate, $m$ is the finite migration rate, $\lambda$ is the finite growth rate, and $G$ is the logistic growth equation."
- Based on observations of negative density-dependent schistosome infection in snails, Gurarie et al. (referenced in the methods) formulated the force of infection as a saturating function $\Lambda = \Lambda^*(1 - e^{-\beta w})$, where the force of infection at endemic equilibrium (without treatment) is at its maximum ($\Lambda^*$). The force of infection, $\Lambda$, is dynamic, but it does not increase linearly with worm burden $w$ as simple models have assumed. Gurarie et al. used this expression because a force of infection that increases linearly with worm burden, as a term $\beta w$ assumes, does not capture equilibrium and out-of-equilibrium dynamics of schistosome infection in humans given the same set of parameter values. We used their framework to, for example, more accurately describe the rebound of human infections observed after treatment that is not explained by change in human immunity and may be best explained by negative density-dependent schistosome infection in snails.
- The calculation of parameters using the negative binomial distribution and discussion about the implications of a stationary value for k are found in pages 36-40 of the supplement and table S2. We agree that estimating k can be challenging due to the fact that in some settings k is not stationary in a treated human population as prevalence of infection declines. The value for k used in our manuscript is estimated for endemic *S. mansoni*, however it is correct that some variation in model results could exist for different populations. We have added reference to SI Table 2 in the caption of figure 1. We have elaborated the definition of $\beta$ in the methods (lines 111-112) to make it clear how the distribution of adult worms in the human population is incorporated in the model (lines 327-330): "$\beta$ is a function of the shape of the negative binomial distribution of adult worms in the human population (SI Table 2)." We have also included discussion on variation of the results due to this parameter and others: "Our analyses primarily explored variation in GDMI success due to genetic parameters, but epidemiological parameters, such as the shape of the distribution of adult worms in the human population or the lifespan of the snails in a local environment, can also influence these results."
- The genetic model validation we performed was specific to *B. glabrata* since this species is the only snail host for which the genetics for immunity have been well characterized. We clarify as suggested in lines 155-157: "The values of some genetic model parameters may differ for other snail species or genes conferring immunity than those so far studied for *B. glabrata*."
- We agree that a table of parameters is essential and have included the two tables for genetic and epidemiological parameters along with their source or derivation in the supplement.

Eliminating many parameters from these tables to construct a key parameters table for the main text would be challenging, and in our opinion, allowing readers to refer to these supplemental tables gives a fuller understanding of the model. We have included additional references to these tables in the main text, which can now be found on lines 112, 119, 167, 250 and the caption of figure 1.

- These are good suggestions to improve figure 2, and we have incorporated them. The titles and y-axes are changed, the empirical data is represented with points, and each point has error bars representing 95% CI calculated from binomial standard error ($1.96 * \sqrt{p(1-p)/n}$). The caption is modified to explain the error bars. Because we assumed mating in this experiment was primarily through outcrossing due to proximity of mates, selfing, and therefore, inbreeding costs, were not simulated in the model results in figure 2. For this reason, varying the inbreeding costs to produce CI around the model values is not possible.
- We recognize the terminology is not mathematically precise for 'selfing rate', and in fact, early drafts of our manuscript used 'selfing frequency' instead, which is equivalent to 'selfing probability'. However, we changed the wording to 'selfing rate' because it is convention in the quantitative genetics literature to refer to the probability or frequency of selfing as 'selfing rate', which is ambiguous but not inaccurate if we consider the rate to be selfing per fertilization event.
- We agree that adjusting fig. 3b to differentiate the color of the simulation from lines in fig. 3a is helpful and have done so. The simulation, confidence interval, and vertical dotted line are now a shade of green. The bars representing the range of selfing values by snail species are colored based on genus to visually group relatives and demonstrate that selfing rate can vary significantly with close relatives.
- While we agree that a few of the parameters explored could be correlated in some settings, it is also likely that in other settings they will be uncorrelated. One could envision constraints on the simultaneous production of modified snails, which would lend to multiple introductions over time rather than a bulk introduction. The speed of gene drive fixation in the population will determine the strategy used to optimize the release of one large or several smaller modified 'seeds'. We suspect that as the field matures, more detailed analyses of this type of optimization may be important and have chosen to not constrain the analysis at this early modeling stage. Observing the separate impact of each parameter could be valuable for future studies.
- This is a good clarification, and we do so in line 251 to state that the "prevalence of infection in humans is assumed to be 80%". In high transmission areas this is a commonly observed prevalence in humans. The mean worm burden is calculated directly from this endemic prevalence given k=0.24 for the negative binomial distribution of worms in a human population (details in the supplement).
- The epidemiological results could be elaborated in the abstract as suggested, and we have included the sentence: "Alone a drive modifying snail immunity could achieve significant disease reduction in humans several years after release." We have also provided an additional figure panel to SI figure 13 simulating conditions in which GDMI is deployed in a treated human population. The human population is treated for five years prior to GDMI release. We update the caption and provide language at the end of the supplement describing these results: "In a human population previously treated with MDA to which GDMI is later added, similar patterns to a previously untreated population emerge but on different timelines. With MDA alone, MWB stablizes to a lower level than without treatment. Adding GDMI to this treatment regime leads to long-term elimination. Elimination is achieved with GDMI alone, but in the short-term after MDA is discontinued, MWB increases for a decade before falling again. Elimination is contingent on the continued success of GDMI over decades, which could be challenged by the evolution of resistance to GDMI or extinction, as discussed in the main text and supplement." We have included a sentence

referencing these results in the main text as well (lines 258-259): "This is true for previously MDA treated and untreated populations (Fig. S13)."

- In lines 261-262, we mention: "Moreover, immunity in snails wanes due to decreased force of infection on the snail population, resulting in an upward trend in MWB from year five onward."
- We have written out the full wording for the acronym in the figure 5 caption, and this can be found additionally in line 247.
- Naturally occurring immune snails can be fully or partially immune. Based on the majority of experimental evidence, penetrance of immunity is approximately 80%. This is the value we used in the analyses, which we have indicated in SI Table 1. We assumed the same penetrance for GDMI, though developed constructs will require evaluation.

Thank you again for the manuscript improvements. We hope you find that we have diligently incorporated them to the best of our abilities and that the manuscript is satisfactory for publication.

We send our best regards,

Dr. R. Grewelle, Dr. J. Perez-Saez, Dr. J. Tycko, Dr. E. Namigai, C. Rickards, Dr. G. De Leo
